# Bayesian comparison of explicit and implicit causal inference strategies in multisensory heading perception

**DOI:** 10.1371/journal.pcbi.1006110

**Published:** 2018-07-27

**Authors:** Luigi Acerbi, Kalpana Dokka, Dora E. Angelaki, Wei Ji Ma

**Affiliations:** 1 Center for Neural Science, New York University, New York, NY, United States of America; 2 Department of Neuroscience, Baylor College of Medicine, Houston, TX, United States of America; 3 Department of Psychology, New York University, New York, NY, United States of America; Harvard University, UNITED STATES

## Abstract

The precision of multisensory perception improves when cues arising from the same cause are integrated, such as visual and vestibular heading cues for an observer moving through a stationary environment. In order to determine how the cues should be processed, the brain must infer the causal relationship underlying the multisensory cues. In heading perception, however, it is unclear whether observers follow the Bayesian strategy, a simpler non-Bayesian heuristic, or even perform causal inference at all. We developed an efficient and robust computational framework to perform Bayesian model comparison of causal inference strategies, which incorporates a number of alternative assumptions about the observers. With this framework, we investigated whether human observers’ performance in an *explicit* cause attribution and an *implicit* heading discrimination task can be modeled as a causal inference process. In the explicit causal inference task, all subjects accounted for cue disparity when reporting judgments of common cause, although not necessarily all in a Bayesian fashion. By contrast, but in agreement with previous findings, data from the heading discrimination task only could not rule out that several of the same observers were adopting a forced-fusion strategy, whereby cues are integrated regardless of disparity. Only when we combined evidence from both tasks we were able to rule out forced-fusion in the heading discrimination task. Crucially, findings were robust across a number of variants of models and analyses. Our results demonstrate that our proposed computational framework allows researchers to ask complex questions within a rigorous Bayesian framework that accounts for parameter and model uncertainty.

## Author summary

As we interact with objects and people in the environment, we are constantly exposed to numerous sensory stimuli. For safe navigation and meaningful interaction with entities in the environment, our brain must determine if the sensory inputs arose from a common or different causes in order to determine whether they should be integrated into a unified percept. However, how our brain performs such a causal inference process is not well understood, partly due to the lack of computational tools that can address the complex repertoire of assumptions required for modeling human perception. We have developed a set of computational algorithms that characterize the causal inference process within a quantitative model based framework. We have tested the efficacy of our methods in predicting how human observers judge visual-vestibular heading. Specifically, our algorithms perform rigorous comparison of alternative models of causal inference that encompass a wide repertoire of assumptions observers may have about their internal noise or stimulus statistics. Importantly, our tools are widely applicable to modeling other processes that characterize perception.

## Introduction

We constantly interact with people and objects around us. As a consequence, our brain receives information from multiple senses as well as multiple inputs from the same sense. Cues from the same sense (e.g., texture and disparity cues to an object shape) are generally congruent as they usually reflect identical properties of a common external entity. Thus, the brain eventually learns to mandatorily integrate inputs from the same modality as a unified percept, which provides more precise information than either cue alone [1, 2]. Similarly, integration of cues represented in different modalities but associated with a common stimulus also improves perceptual behavior. There is a wealth of evidence that demonstrates increased precision [3–12], greater accuracy [13, 14] and faster speed [15, 16] of perceptual performance due to multimodal integration.

However, multimodal cues present a complex problem. Cues from different modalities are not necessarily congruent as different stimuli can simultaneously impinge on our senses, giving rise to coincident yet conflicting information. For example, in a classic ventriloquist illusion, even though the sound originates from the puppeteer’s mouth, we perceive that it is the puppet which is talking [17]. Mandatory integration of multimodal cues arising from different stimuli can induce errors in perceptual estimates [6, 14]. Thus, for efficient interaction with the world, the brain must assess whether the multimodal cues originated from the same cause, and should be integrated into a single percept, or instead the cues should be interpreted in isolation as they arose from different causes (segregation). Despite the often overwhelming amount of sensory inputs, we are typically able to integrate relevant cues while ignoring irrelevant sensory input. It is thus plausible that our brain infers the causal relationship between multisensory cues to determine if and how the cues should be integrated.

Bayesian causal inference—inference of the causal relationship between observed cues, based on the inversion of the statistical model of the task—has been proposed as the decision strategy adopted by the brain to address the problem of integration vs. segregation of sensory cues [18, 19]. Such a decision strategy has described human performance in spatial localization [18–27], orientation judgment [28], oddity detection [29], speech perception [30], time-interval perception [31], simple perceptual organization [32], and heading perception [33, 34]. In recent years, interest in the Bayesian approach to causal inference has further increased as neural imaging has identified a hierarchy of brain areas involved in neural processing while observers implemented a Bayesian strategy to perform a causal inference task [20]. At the same time, Bayesian models have become more complex as they include more precise descriptions of the sensory noise [22, 33, 34] and alternative Bayesian decision strategies [21, 24]. However, it is still unknown whether observers fully implement Bayesian causal inference, or merely an approximation that does not take into account the full statistical structure of the task. For example, the Bayes-optimal inference strategy ought to incorporate sensory uncertainty into its decision rule. On the other hand, a suboptimal heuristic decision rule may disregard sensory uncertainty [32, 35, 36]. Thus, the growing complexity of models and the need to consider alternative hypotheses require an efficient computational framework to address these open questions while avoiding trappings such as overfitting or lack of model identifiability [37]. For a more detailed overview of open issues in multisensory perception and causal inference at the intersection of behavior, neurophysiology and computational modeling, we refer the reader to [38–40].

### Visuo-vestibular integration in heading perception

Visuo-vestibular integration in heading perception presents an ideal case to characterize the details of the causal inference strategy in multisensory perception. While a wealth of published studies have shown that integration of visual and vestibular self-motion cues increases perceptual precision [9–12, 14, 41–43], and accuracy [14], such an integration only makes sense if the two cues arise from the same cause—that is optic flow and inertial motion signal heading in the same direction. Despite the putative relevance of causal inference in heading perception, the inference strategies that characterize visuo-vestibular integration in the presence of sensory conflict remain poorly understood. For example, a recent study has found that observers predominantly integrated visual and vestibular cues even in the presence of large spatial discrepancies [33]—whereas a subsequent work has presented evidence in favor of causal inference [34]. Furthermore, these studies did not vary cue reliability—a manipulation that is critical to test whether a Bayes-optimal inference strategy or a suboptimal approximation was used [35].

Another aspect that can influence the choice of inference strategy is the type of inference performed by the observer. In particular, de Winkel and colleagues [33, 34] asked subjects to indicate the perceived direction of inertial heading—an ‘implicit’ causal inference task as subjects implicitly assessed the causal relationship between visual and vestibular cues on their way to indicate the final (integrated or segregated) heading percept. Even in the presence of spatial disparities as high as 90°, one study found that several subjects were best described by a model which fully integrated visual and vestibular cues [33] (possibly influenced by the experimental design; see also [34]). It is plausible that performing an explicit causal inference task, which forces subjects to indicate whether visual and vestibular cues arose from the same or different events, may elicit different inference strategies, as previously reported in category-based induction [44], multi-cue judgment [45], and sensorimotor decision-making [46]. While some studies have tested both explicit and implicit causal inference [18, 21, 47], to our knowledge only one previous study contemplated the possibility of different strategies between implicit and explicit causal inference tasks [21], and a systematic comparison of inference strategies in the two tasks has never been carried out within a larger computational framework.

### Bayesian comparison of causal inference strategies

Thus, the goal of this work is two-fold. First, we introduce a set of techniques to perform robust, efficient Bayesian factorial model comparison of a variety of Bayesian and non-Bayesian models of causal inference in multisensory perception. Factorial comparison is a way to simultaneously test different orthogonal hypotheses about the observers [21, 48–50]. Our approach is fully Bayesian in that we consider both parameter and model uncertainty, improving over previous analyses which used point estimates for the parameters and compared individual models. A full account of uncertainty in both parameter and model space, by marginalizing over parameters and model components, is particularly prudent when dealing with internal processes, such as decision strategies, which may have different latent explanations. An analysis that disregards such uncertainty might produce unwarranted conclusions about the internal processes that generated the observed behavior [37]. Second, we demonstrate our methods by quantitatively comparing the decision strategies underlying explicit and implicit causal inference in visuo-vestibular heading perception within the framework of Bayesian model comparison. We found that even though the study of explicit and implicit causal inference in isolation might suggest different inference rules, a joint analysis that combines all available evidence points to no difference between tasks, with subjects performing some form of causal inference in both the explicit and implicit tasks that used identical experimental setups.

In sum, we demonstrate how state-of-the-art techniques for model building, fitting, and comparison, combined with advanced analysis tools, allow us to ask nuanced questions about the observer’s decision strategies in causal inference. Importantly, these methods come with a number of diagnostics, sanity checks and a rigorous quantification of uncertainty that allow the experimenter to be explicit about the weight of evidence.

## Results

### Computational framework

We compiled a diverse set of computational techniques to perform robust Bayesian comparison of models of causal inference in multisensory perception, which we dub the ‘Bayesian cookbook for causal inference in multisensory perception’, or herein simply ‘the cookbook’. The main goal of the cookbook is to characterize observers’ decision strategies underlying causal inference, and possibly other details thereof, within a rigorous Bayesian framework that accounts for both parameter uncertainty and model uncertainty. The cookbook is ‘doubly-Bayesian’ in that it affords a fully Bayesian analysis of observers who may or may not be performing Bayesian inference themselves [51]. Fully Bayesian model comparison is computationally intensive, hence the cookbook is concerned with efficient algorithmic solutions.

The cookbook comprises of: (a) a fairly general recipe for building observer models for causal inference in multisensory perception (see Methods and Section 1 of S1 Appendix), which lends itself to a factorial model comparison; (b) techniques for fast evaluation of a large number of causal inference observer models; (c) procedures for model fitting via maximum likelihood, and approximating the Bayesian posterior of the parameters via Markov Chain Monte Carlo (MCMC); (d) state-of-the-art methods to compute model comparison metrics and perform factorial model selection. It is noteworthy that, while the current work focuses on the example of visuo-vestibular heading perception, this cookbook is general and can be applied with minor modifications to multisensory perception across sensory domains. Computational details are described in the Methods section and S1 Appendix. Here we present an application of our framework to causal inference in multisensory heading perception. For ease of reference, we summarize relevant abbreviations used in the paper and their meaning in Table 1.

**Table 1:** Abbreviations and symbols.

Abbreviation	Meaning	Context
	**General**	
Δ	Directional disparity between stimuli	Generative model
*s*_vis_, *s*_vest_	Visual / vestibular heading	Generative model
*x*_vis_, *x*_vest_	Noisy measurement of visual / vestibular heading	Generative model
*C*	Causal scenario (*C* = 1 for ‘same’, *C* = 2 for ‘different’)	Generative model
*c*_vis_	Visual coherence level (low, medium, or high)	Generative model
*p*_c_	Probability of common cause (Bayesian model)	Observer model
*κ*_c_	Criterion for common cause (fixed-criterion model)	Observer model
	**Model factors**	
Bay	Bayesian strategy	Causal inference strategy
Fix	Fixed-criterion strategy	Causal inference strategy
Fus	Fusion strategy	Causal inference strategy
-C	Constant noise	Sensory noise shape
-X	Eccentricity-dependent noise	Sensory noise shape
-E	Empirical prior	Prior type
-I	Independent priors	Prior type
	**Model fitting and comparison**	
AIC(c)	(corrected) Akaike’s Information Criterion	Model comparison metric
BIC	Bayesian Information Criterion	Model comparison metric
LML	Log marginal likelihood	Model comparison metric
LOO	Leave-one-out	Model comparison metric
MCMC	Markov Chain Monte Carlo	Model fitting technique
$\tilde{\varphi }$	Protected exceedance probability	Bayesian model selection statistic
BOR	Bayesian Omnibus Risk	Bayesian model selection statistic

List of abbreviations and symbols used in the paper, with associated description and usage context.

### Causal inference in heading perception

We demonstrate our framework taking as a case study the comparison of explicit vs. implicit causal inference strategies in heading perception. In this section we briefly summarize our methods. Extended details and description of the cookbook can be found in the Methods and S1 Appendix.

#### Experiments

**Fig 1:**
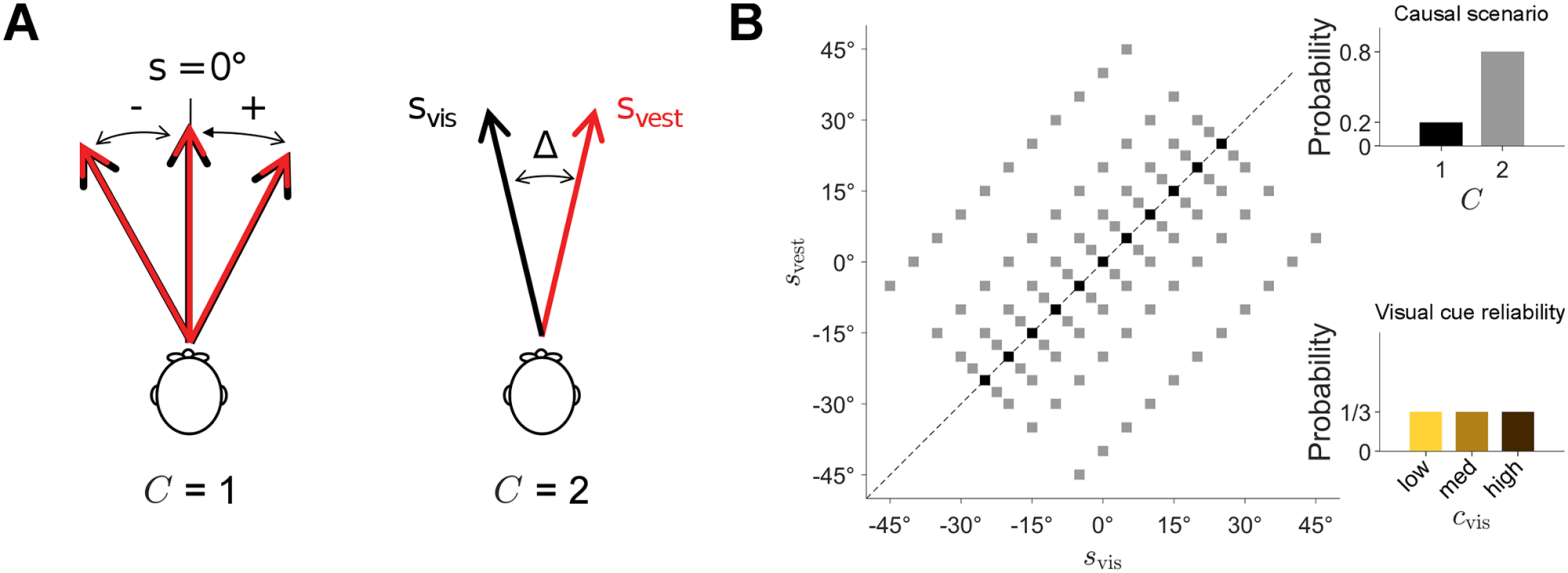
Experiment layout. **A**: Subjects were presented with visual (*s*_vis_) and vestibular (*s*_vis_) headings either in the same direction (*C* = 1) or in different directions (*C* = 2). In different sessions, subjects were asked to judge whether stimuli had the same cause (‘unity judgment’, explicit causal inference) or whether the vestibular heading was to the left or right of straight forward (‘inertial discrimination’, implicit causal inference). **B**: Distribution of stimuli used in the task. Mean stimulus direction was drawn from a discrete uniform distribution (−25°, −20°, −15°,…,25°). In 20% of the trials, *s*_vis_ ≡ *s*_vest_ (‘same’ trials, *C* = 1); in the other 80% (‘different’, *C* = 2), disparity was drawn from a discrete uniform distribution (±5°, ±10°, ±20°, ±40°), which led to a correlated pattern of heading directions *s*_vis_ and *s*_vest_. Visual cue reliability *c*_vis_ was also drawn randomly on each trial (high, medium, and low).

Human observers were presented with synchronous visual (*s*_vis_) and vestibular (*s*_vest_) headings in the same direction (*C* = 1) or in different directions (*C* = 2) separated by a directional disparity Δ (Fig 1A). Mean stimulus direction (−25°, −20°, −15°,…,25°), cue disparity (0°, ±5°, ±10°, ±20°, and ±40°), and visual cue reliability *c*_vis_ (coherence: high, medium and low) changed randomly on a trial-by-trial basis (Fig 1B). On each trial, non-zero disparity was either positive (vestibular heading to the right of visual heading) or negative. Observers (*n* = 11) first performed several sessions of an *explicit* causal inference task (‘unity judgment’), in which they indicated if the visual and vestibular stimuli signaled heading in the *same* direction (‘common cause’) or in *different* directions (‘different causes’). The same observers then participated in a number of sessions of the *implicit* causal inference task (‘inertial left/right discrimination’) wherein they indicated if their perceived inertial heading (vestibular) was to the left or right of straight forward. Both tasks consisted of a binary classification (same/different or left/right) with identical experimental apparatus and stimuli. No feedback was given to subjects about the correctness of their response. All observers also performed a number of practice trials and an initial session of a ‘unisensory left/right discrimination’ task in which they reported heading direction (left or right of straight forward) of visual or vestibular stimuli presented in isolation. For each subject we obtained 350–750 trials of the unisensory discrimination task (1 session), 700-1200 trials of the unity judgment task (2-3 sessions), and 2100-3000 trials of the inertial discrimination task (7-9 sessions).

#### Theory

**Fig 2:**
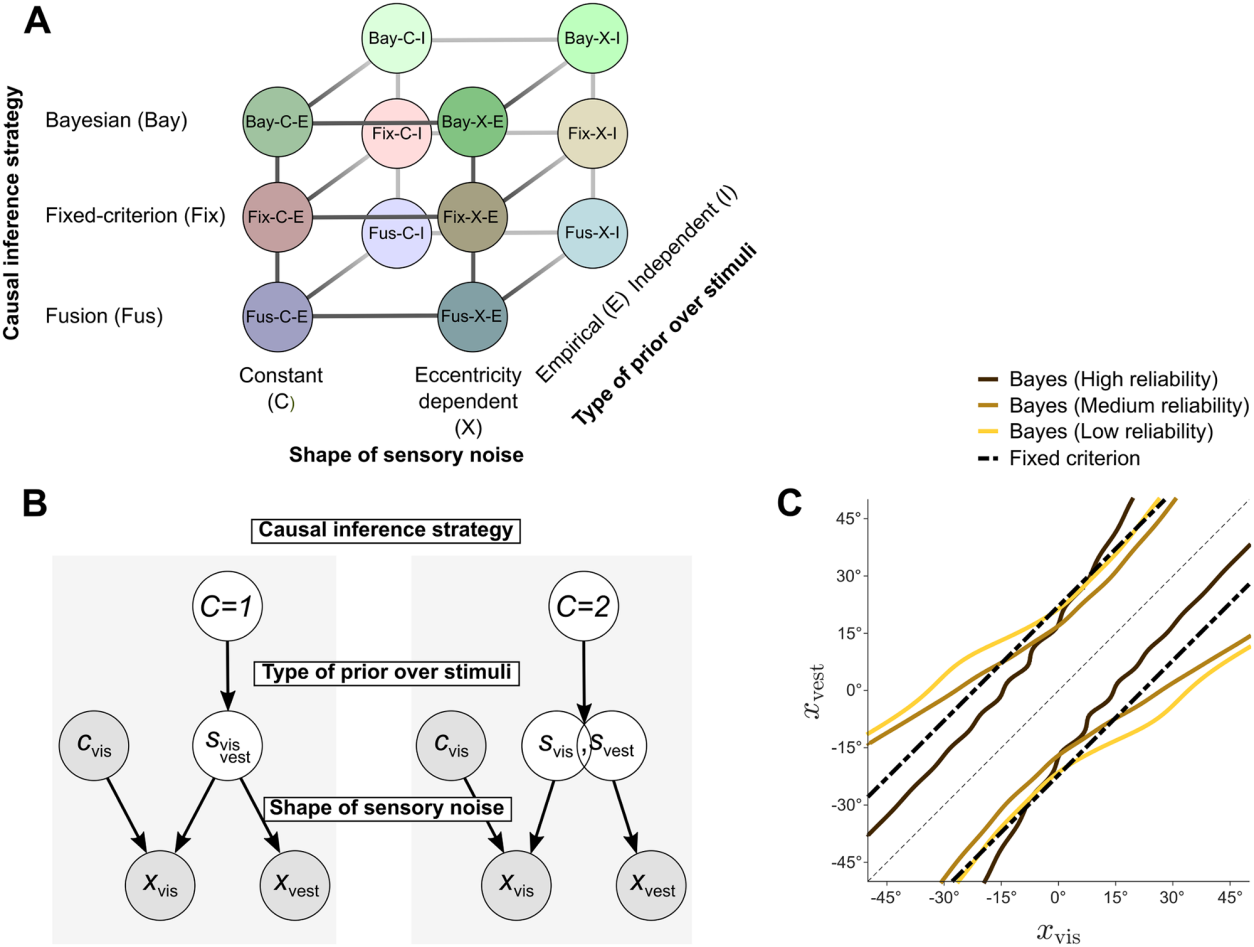
Observer models. **A**: Observer models consist of three model factors: Causal inference strategy, Shape of sensory noise, and Type of prior over stimuli (see text). **B**: Graphical representation of the observer model. In the left panel (*C* = 1), the visual (*s*_vis_) and vestibular (*s*_vest_) heading direction have a single, common cause. In the right panel (*C* = 2), *s*_vis_ and *s*_vest_ have separate sources, although not necessarily statistically independent. The observer has access to noisy sensory measurements *x*_vis_, *x*_vest_, and knows the visual reliability level of the trial *c*_vis_. The observer is either asked to infer the causal structure (unity judgment, explicit causal inference), or whether the vestibular stimulus is rightward of straight ahead (inertial discrimination, implicit causal inference). Model factors affect different stages of the observer model: the strategy used to combine the two causal scenarios; the type of prior over stimuli *p*_prior_(*s*_vis_, *s*_vest_|*C*); and the shape of sensory noise distributions *p*(*x*_vis_|*s*_vis_, *c*_vis_) and *p*(*x*_vest_|*s*_vest_) (which affects equally both how noisy measurements are generated and the observer’s beliefs about such noise). **C**: Example decision boundaries for the Bay-X-E model (for the three reliability levels), and for the Fix model, for a representative observer. The observer reports ‘unity’ when the noisy measurements *x*_vis_, *x*_vest_ fall within the boundaries. Note that the Bayesian decision boundaries expand with larger noise. Nonlinearities are due to the interaction between eccentricity-dependence of the noise and the prior (wiggles are due to the discrete empirical prior).

For each task we built a set of observer models by factorially combining three model components—hence also called model factors—that represent different assumptions about the observers: shape of sensory noise, type of prior over stimuli, and causal inference strategy (Fig 2A).

In each trial of the explicit and implicit causal inference tasks, two stimuli are presented: a visual heading *s*_vis_ with known reliability *c*_vis_ ∈ {high, medium, low}, and a vestibular heading *s*_vest_. We assume that stimuli *s*_vis_, *s*_vest_ induce noisy measurements *x*_vis_ (resp., *x*_vest_) with conditionally independent distributions *p*(*x*_vis_|*s*_vis_, *c*_vis_) and *p*(*x*_vest_|*s*_vest_). For any stimulus *s* we assume that the noise distribution is a (wrapped) Gaussian centered on *s* and with variance *σ*^2^(*s*). For each observer model we consider a variant in which *σ*^2^ depends only on the stimulus modality and reliability (*constant*, ‘C’) and a variant in which *σ*^2^(*s*) also depends on stimulus location, growing with heading eccentricity, that is with the distance from 0° (*eccentricity-dependent*, ‘X’; see Methods). With a few notable exceptions [22, 33, 34], stimulus-dependence in the noise has been generally ignored in previous work [18, 20, 21, 24, 27]. The base noise magnitude is governed by model parameters *σ*_0vest_ and *σ*_0vis_(*c*_vis_), where the latter is one parameter per visual reliability level. The eccentricity-dependent noise model has additional parameters *w*_vest_ and *w*_vis_ which govern the growth of noise with heading eccentricity (see Methods and S1 Appendix for details). We assume that the noise distribution equally affects both the generative model and the observer’s decision model, that is, observers have an approximately correct model of their own sensory noise [4, 6, 9].

We assume that the observer considers two causal scenarios [18]: either there is a single common heading direction (*C* = 1) or the two stimuli correspond to distinct headings (*C* = 2) [18] (Fig 2B). If *C* = 1, the observer believes that the measurements are generated from the same underlying source *s* with prior distribution *p*_prior_(*s*). If *C* = 2, stimuli are believed to be distinct, but not necessarily statistically independent, with prior distribution *p*_prior_(*s*_vis_, *s*_vest_). For the type of these priors, we consider an *empirical* (‘E’) observer whose priors correspond to an approximation of the discrete, correlated distribution of stimuli in the task (as per Fig 1B); and an *independent* (‘I’) observer who uses a common and independent uni-dimensional Gaussian prior centered on 0° for the two stimuli.

Parameter *σ*_prior_ represents the SD of each independent prior (for ‘I’ priors), or of the prior over mean stimulus direction (for ‘E’ priors); whereas Δ_prior_ governs the SD of the prior over disparity (‘E’ priors only). See Methods for details.

We assume that observers are Bayesian in dealing with each causal scenario (*C* = 1 or *C* = 2), but may follow different strategies for weighting and combining information from the two causal hypotheses. Specifically, we consider three families of causal inference strategies. The Bayesian (‘Bay’) strategy computes the posterior probability of each causal scenario Pr(*C*|*x*_vis_, *x*_vest_, *c*_vis_) based on all information available in the trial. The fixed-criterion (‘Fix’) strategy decides based on a fixed threshold of disparity between the noisy visual and vestibular measurements, disregarding reliability and other statistics of the stimuli. Finally, the fusion (‘Fus’) strategy disregards any location information, either always combining cues, or combining them with some probability (depending on whether the task involves implicit or explicit causal inference).

In the explicit causal inference task, the Bayesian (‘Bay’) observer reports a common cause if its posterior probability is greater than 0.5, Pr(*C* = 1|*x*_vis_, *x*_vest_, *c*_vis_) > 0.5. The prior probability of common cause, *p*_c_ ≡ Pr(*C* = 1), is a free parameter of the model. The fixed-criterion (‘Fix’) observer reports a common cause whenever the two noisy measurements are closer than a fixed distance *κ*_c_, that is |*x*_vis_ − *x*_vest_| < *κ*_c_, where the criterion *κ*_c_ is a free parameter that does not depend on stimulus reliability [36]. The fixed-criterion decision rule differs fundamentally from the Bayesian one in that it does not take cue reliability and other stimulus statistics into account (although noise will still affect behavior). As an example, Fig 2C shows the decision boundaries for the Bayesian (constant noise, empirical prior) and fixed-criterion rule for a representative observer. Finally, as a variant of the ‘fusion’ strategy we consider an observer that does not perform causal inference at all, but simply reports unity with probability *η*(*c*_vis_) regardless of stimulus disparity, where *η*_low_, *η*_med_, *η*_high_ are the only parameters of the model (*stochastic fusion*, ‘SFu’). This variant generalizes a trivial ‘forced fusion’ strategy (*η* ≡ 1) that would always report a common cause in the explicit inference.

For the implicit causal inference task, the observer first computes the posterior probability of rightward vestibular motion, Pr(*s*_vest_ > 0°|*x*_vest_, *x*_vis_, *c*_vis_, *C* = *k*) for the two causal scenarios, *k* = 1, 2. The Bayesian (‘Bay’) observer then reports ‘right’ if the posterior probability of rightward vestibular heading, averaged over the Bayesian posterior over causal structures, is greater than 0.5. The fixed-criterion (‘Fix’) observer reports ‘right’ if Pr(*s*_vest_ > 0°|*x*_vest_, *x*_vis_, *c*_vis_, *C* = *k*_fix_) > 0.5, where *k*_fix_ = 1 if |*x*_vis_ − *x*_vest_| < *κ*_c_, and *k*_fix_ = 2 otherwise. Finally, for the Fusion strategy we consider here the *forced fusion* (‘FFu’) observer, for which *C* ≡ 1. The forced fusion observer is equivalent to a Bayesian observer with *p*_c_ ≡ 1, and to a fixed-criterion observer for *κ*_c_ → ∞.

Observers also performed a unisensory left/right heading discrimination task, in which either a visual or vestibular heading was presented on each trial. In this case observers were modeled as standard Bayesian observers that respond ‘right’ if Pr(*s*_vis_ > 0°|*x*_vis_, *c*_vis_) > 0.5 for visual trials, and if Pr(*s*_vest_ > 0°|*x*_vest_) > 0.5 for vestibular trials. These data were used to constrain the joint model fits (see below).

For all observer models and tasks (except stochastic fusion in the explicit task), we considered a lapse probability 0 ≤ λ ≤ 1 of the observer giving a random response. Finally, we note that the Bayesian observer models considered in our main analysis perform Bayesian model averaging (the proper Bayesian strategy). At the end of the Results section we will also consider a ‘probability matching’ suboptimal Bayesian observer [24].

#### Analysis strategy

Our analysis strategy consisted of first examining subjects’ behavior separately in the explicit and implicit tasks via model fitting and comparison. We then compared the model fits across tasks to ensure that model parameters were broadly compatible, allowing us to aggregate data from different tasks without changing the structure of the models. Finally, we re-analyzed observers’ performance by jointly fitting data from all three tasks (explicit causal inference, implicit causal inference, and unisensory heading discrimination), thereby combining all available evidence to characterize subjects’ decision making processes.

Given the large number of models and distinct datasets involved, we coded each model using efficient computational techniques at each step (see Methods for details).

We fitted our models to the data first via maximum-likelihood estimation, and then via Bayesian estimation of the posterior over parameters using Markov Chain Monte Carlo (MCMC). Posteriors are an improvement over point estimates in that they allow us to incorporate uncertainty over individual subjects’ model parameters in our analysis, and afford computation of more accurate comparison metrics (see below).

We computed for each task, subject, and model the leave-one-out cross-validation score (LOO) directly estimated from the MCMC output [52] (reported in S1 Appendix). LOO has several advantages over other model selection metrics in that it takes parameter uncertainty into account and provides a more accurate measure of predictive performance [53] (see Discussion). We combined model evidence (LOO scores) from different subjects and models using a hierarchical Bayesian approach for group studies [54]. For each model component within the model factors of interest (noise, prior, and causal inference strategy), we reported as the main summary statistic of the analysis the protected exceedence probability $\tilde{\varphi }$, that is the (posterior) probability of a model component being the most likely component, above and beyond chance [55]. As a test of robustness, we also computed additional model comparison metrics: the corrected Akaike’s information criterion (AICc), the Bayesian information criterion (BIC), and an estimate of the log marginal likelihood (LML). While we prefer LOO as the main metric (see Discussion), we verified that the results of the model comparison were largely invariant of the choice of comparison metric.

Finally, for each model we estimated the absolute goodness of fit as the fraction of information gain above chance (where 0% is chance and 100% is the estimated intrinsic variability of the data, that is the entropy [56]).

### Explicit causal inference task

**Fig 3:**
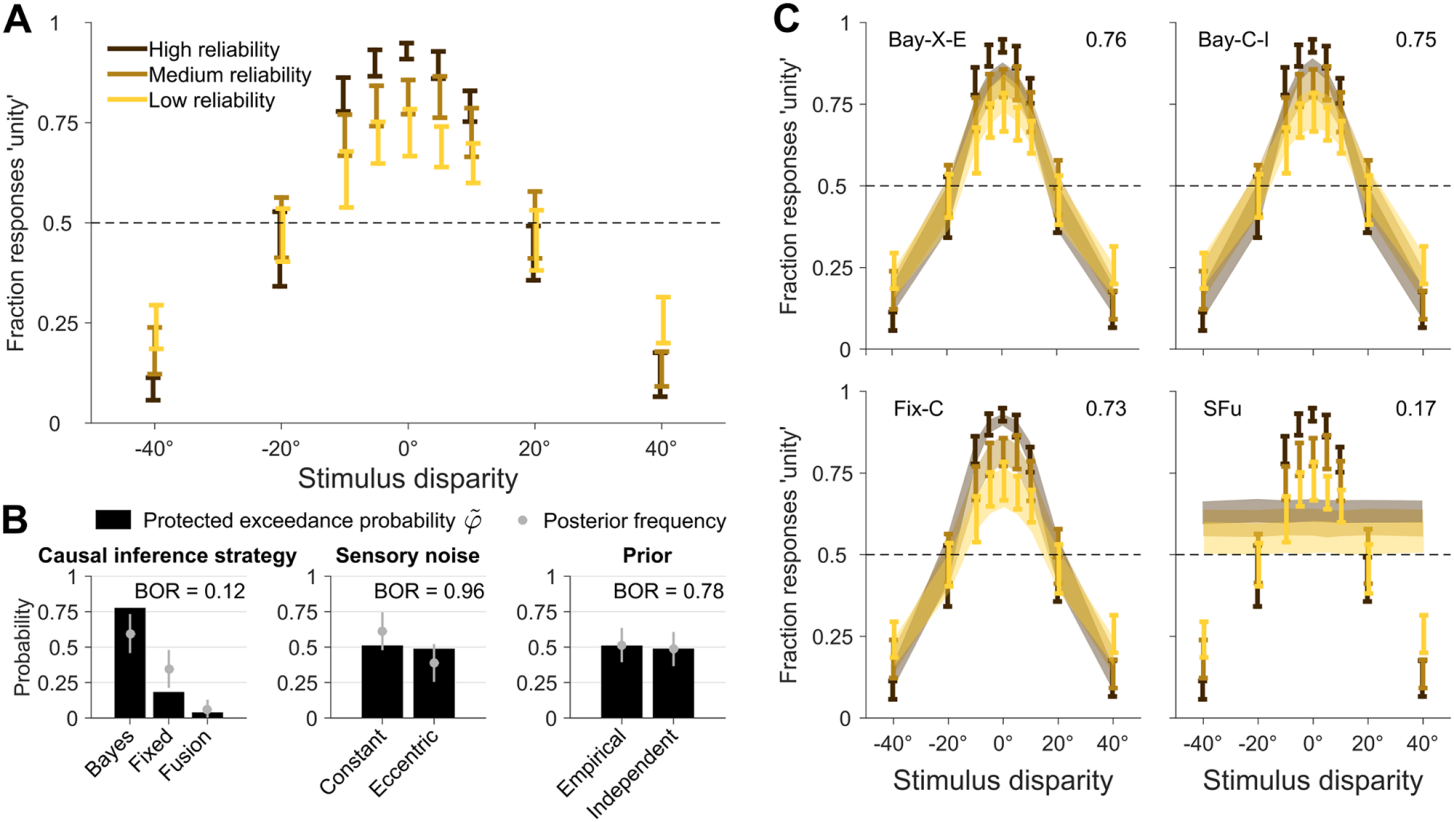
Explicit causal inference. Results of the explicit causal inference (unity judgment) task. **A**: Proportion of ‘unity’ responses, as a function of stimulus disparity (difference between vestibular and visual heading direction), and for different levels of visual cue reliability. Bars are ±1 SEM across subjects. Unity judgments are modulated by stimulus disparity and visual cue reliability. **B**: Protected exceedance probability $\tilde{\varphi }$ and estimated posterior frequency (mean ± SD) of distinct model components for each model factor. Each factor also displays the Bayesian omnibus risk (BOR). **C**: Model fits of several models of interest (see text for details). Shaded areas are ±1 SEM of model predictions across subjects. Numbers on top right of each panel report the absolute goodness of fit.

We examined how subjects perceived the causal relationship of synchronous visual and vestibular headings as a function of disparity (*s*_vest_ − *s*_vis_, nine levels) and visual reliability level (high, medium, low; Fig 3A). Common cause reports were more frequent near zero disparities than for well-separated stimuli (Repeated-measures ANOVA with Greenhouse-Geisser correction; *F*_(1.82,18.17)_ = 76.0, *ϵ* = 0.23, *p* < 10^−4^, $\eta_{p}^{2}=0.88$). This means that observers neither performed complete integration (always reporting a common cause) nor complete segregation (never reporting a common cause). Common-cause reports were not affected by visual cue reliability alone (*F*_(1.23,12.33)_ = 1.84, *ϵ* = 0.62, *p* = .2, $\eta_{p}^{2}=0.16$), but were modulated by an interaction of visual reliability and disparity (*F*_(7.44,74.44)_ = 7.38, *ϵ* = 0.47, *p* < 10^−4^, $\eta_{p}^{2}=0.42$). Thus, observers’ performance was affected by both cue disparity as well as visual cue reliability when explicitly reporting about the causal relationship between visual and vestibular cues. However, this does not necessarily mean that the subjects’ causal inference strategy took visual cue reliability into account. Changes in sensory noise may affect measured behavior even if the observer’s decision rule ignores such changes [35]; a quantitative model comparison is needed to probe this question.

We compared a subset of models from the full factorial comparison (Fig 2A), since some models are equivalent when restricted to the explicit causal inference task. In particular, here fixed-criterion models are not influenced by the ‘prior’ factor, and the (stochastic) fusion model is not affected by sensory noise or prior, thus reducing the list of models to seven: Bay-C-E, Bay-C-I, Bay-X-E, Bay-X-I, Fix-C, Fix-X, SFu.

To assess the evidence for distinct determinants of subjects’ behavior, we combined LOO scores from individual subjects and models with a hierarchical Bayesian approach [54] (Fig 3B). Since we are investigating model factors that comprise of an unequal number of models, we reweighted the prior over models such that distinct components within each model factor had equal prior probability (Fix models had 2× weight, and SFu 4×). In Fig 3B we report the protected exceedance probabilities $\tilde{\varphi }$ and, for reference, the posterior model frequencies they are based on, and the Bayesian omnibus risk (BOR), which is the estimated probability that the observed differences in factor frequencies may be due to chance [55]. We found that the most likely factor of causal inference was the Bayesian model ($\tilde{\varphi }=0.78$), followed by fixed-criterion ($\tilde{\varphi }=0.18$) and probabilistic fusion ($\tilde{\varphi }=0.04$). That is, fusion was ∼ 24 times less likely to be the most representative model than any form of causal inference combined, which is strong evidence against fusion, and in agreement with our model-free analysis. The Bayesian strategy was ∼ 3.5 times more likely than the others, which is positive but not strong evidence [57]. Conversely, the explicit causal inference data do not allow us to draw conclusions about noise models (constant vs. eccentric) or priors (empirical vs. independent), as we found that all factor components are about equally likely ($\tilde{\varphi }∼0.5$).

At the level of specific models—as opposed to aggregate model factors –, we found that the probability of being the most likely model was almost equally divided between fixed-criterion (C-I) and Bayesian (either X-E or C-I). All these models yielded reasonable fits (Fig 3C), which captured a large fraction of the noise in the data (absolute goodness of fit ≈ 76% ± 3%; see Methods); a large improvement over a constant-probability model, which had a goodness of fit of 14 ± 5%. For comparison, we also show in Fig 3C the stochastic fusion model, which had a goodness of fit of 17 %± 5%. Visually, the Fix model in Fig 3C seems to fit better the group data, but we found that this is an artifact of projecting the data on the disparity axis. Disparity is the only relevant dimension for the Fix model; whereas Bay models fits the data along all dimensions. The visual superiority of the Fix model wanes when the data are visualized in their entirety (see S1 Fig).

We verified robustness of our findings by performing the same hierarchical analysis with different model comparison metrics. All metrics were in agreement with respect to the Bayesian causal inference strategy as the most likely, and the same three models being most probable (although possibly with different ranking). BIC and marginal likelihood differed from LOO and AICc mainly in that they reported a larger probability for the constant vs. eccentricity-dependent noise (probability ratio ∼4.6, which is positive but not strong evidence).

These results combined provide strong evidence that subjects in the explicit causal inference task took into account some elements of the statistical structure of the trial (disparity, and possibly cue reliability) to report unity judgments, consistent with causal inference, potentially in a Bayesian manner. From these data, it is unclear whether observers took into account the empirical distribution of stimuli, and whether their behavior was affected by eccentricity-dependence in the sensory noise.

### Implicit causal inference task

**Fig 4:**
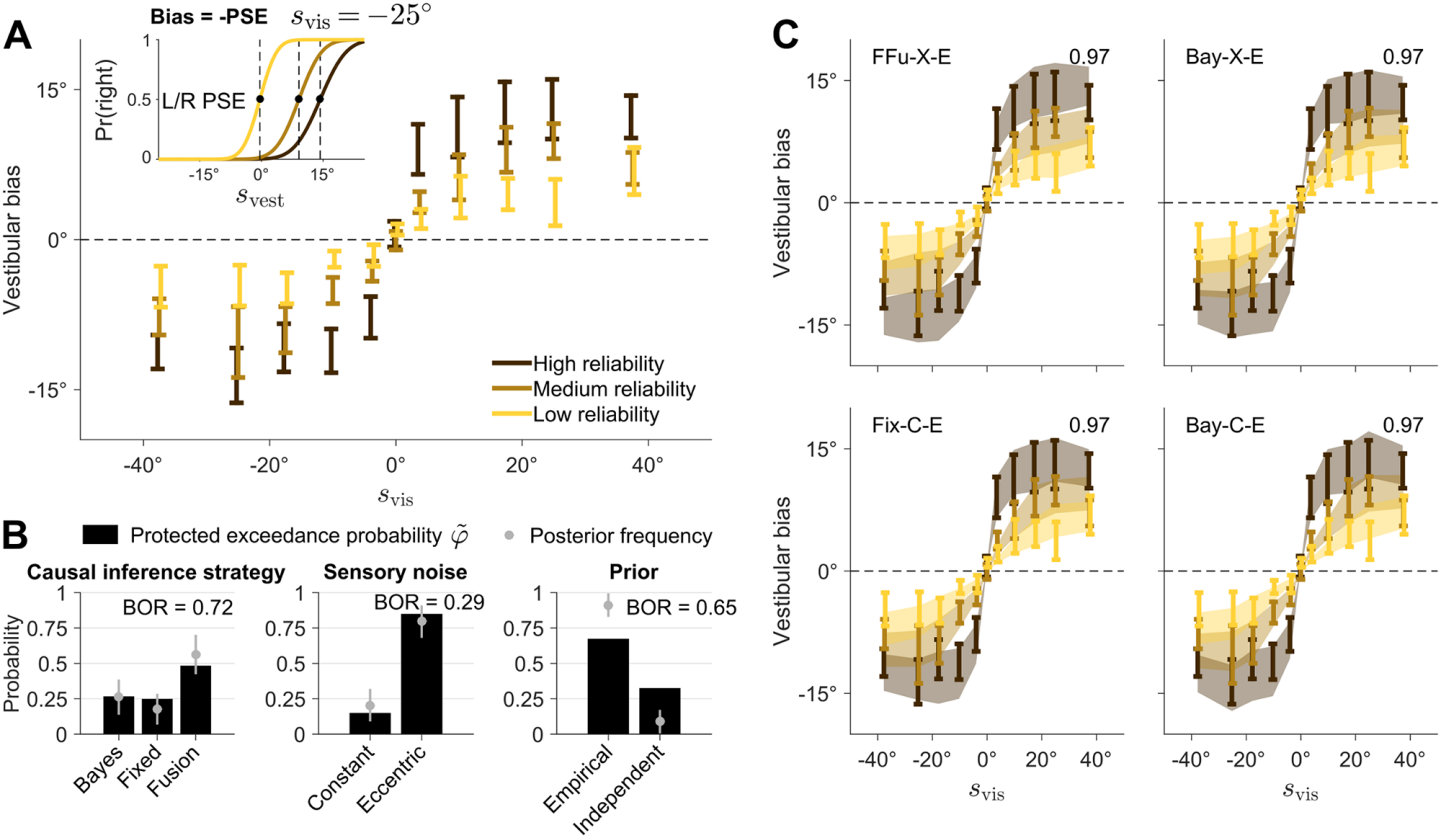
Implicit causal inference. Results of the implicit causal inference (left/right inertial discrimination) task. **A**: Vestibular bias as a function of co-presented visual heading direction *s*_vis_, at different levels of visual reliability. Bars are ±1 SEM across subjects. The inset shows a cartoon of how the vestibular bias is computed as minus the point of subjective equality of the psychometric curves of left/right responses (L/R PSE) for vestibular stimuli *s*_vest_, for a representative subject and for a fixed value of *s*_vis_. The vestibular bias is strongly modulated by *s*_vis_ and its reliability. **B**: Protected exceedance probability $\tilde{\varphi }$ and estimated posterior frequency (mean ± SD) of distinct model components for each model factor. Each factor also displays the Bayesian omnibus risk (BOR). **C**: Model fits of several models of interests (see text for details). Shaded areas are ±1 SEM of model predictions across subjects. Numbers on top right of each panel report the absolute goodness of fit.

We examined the bias in the reported direction of inertial heading computed as (minus) the point of subjective equality for left/rightward heading choices (L/R PSE), for each visual heading and visual cue reliability (Fig 4A). Specifically, for a given value of visual heading *s*_vis_ (or small range thereof), we constructed a psychometric function as a function of *s*_vest_ (see Methods for details). If subjects were influenced by *s*_vis_ and took visual heading into account while computing inertial heading, this would manifest as bias in the psychometric function (that is, a shifted point of subjective equality). If subjects were able instead to discount the distracting influence of *s*_vis_, there should be negligible bias. As per causal inference, we qualitatively expected that there would be bias for smaller |*s*_vis_|, but the bias would either decrease or saturate as |*s*_vis_| increases. However, note that a nonlinear pattern of bias may also emerge due to eccentricity-dependence of the noise, even in the absence of causal inference.

The bias was significantly affected by visual heading (Repeated-measures ANOVA; *F*_(0.71,7.08)_ = 19.67, *ϵ* = 0.07, *p* = .004, $\eta_{p}^{2}=0.66$). We found no main effect of visual cue reliability alone (*F*_(0.85,8.54)_ = 0.51, *ϵ* = 0.43, *p* = .47, $\eta_{p}^{2}=0.05$), but there was a significant interaction of visual cue reliability and heading (*F*_(2.93,29.26)_ = 7.36, *ϵ* = 0.15, *p* < 10^−3^, $\eta_{p}^{2}=0.42$). These data suggest that subjects’ perception of vestibular headings was modulated by visual cue reliability and visual stimulus, in agreement with previous work in visual-auditory localization [21]. However, quantitative model comparison is required to understand the mechanism in detail since distinct processes, such as different causal inference strategies and noise models, could lead to similar patterns of observed behavior.

We performed a factorial comparison with all models in Fig 2A. In this case, factorial model comparison via LOO was unable to uniquely identify the causal inference strategy adopted by observers (Fig 4B). Forced fusion was slightly favored ($\tilde{\varphi }∼0.48$), followed by Bayes ($\tilde{\varphi }∼0.27$) and fixed-criterion ($\tilde{\varphi }∼0.25$), suggesting that all strategies were similar to forced fusion. Conversely, eccentricity-dependent noise was found to be more likely than constant noise (ratio ∼ 5.7), which is positive but not strong evidence, and empirical priors were marginally more likely than independent priors (∼ 2.1). The estimated Bayesian omnibus risk was high (BOR ≥ 0.29), hinting at a large degree of similarity within all model factors such that observed differences could have arisen by chance.

All metrics generally agreed on the lack of evidence in favor of any specific inference strategy (with AICc and BIC tending to marginally favor fixed-criterion instead of fusion), and on empirical priors being more likely. As a notable difference, marginal likelihood and BIC reversed the result about noise models, favoring constant noise models over eccentricity-dependent ones.

In terms of individual models, the most likely models according to LOO were, in order, forced fusion (X-E), Bayesian (X-E), and fixed-criterion (C-E). However, other metrics also favored other models; for example, Bayesian (C-E) was most likely according to the marginal likelihood. All these models obtained similarly good fits to individual data (Fig 4C; absolute goodness of fit ≈ 97%). For reference, a model that responds ‘rightward motion’ with constant probability performed about at chance (goodness of fit ≈ 0.3 ± 0.1%).

**Fig 5:**
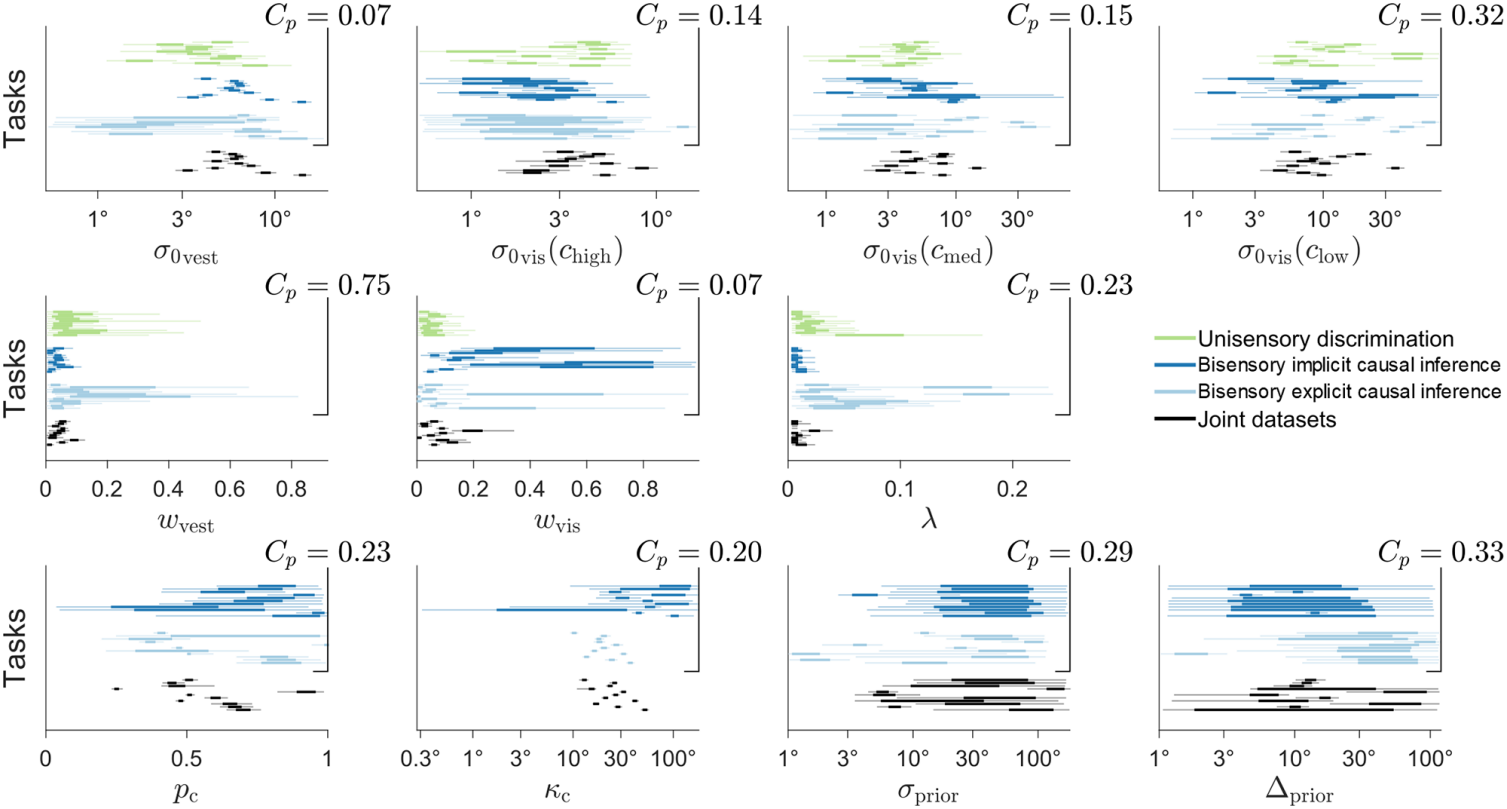
Posteriors over model parameters. Each panel shows the marginal posterior distributions over a single parameter for each subject and task. Each line is an individual subject’s posterior (thick line: interquartile range; light line: 95% credible interval); different colors correspond to different tasks. For each subject and task, posteriors are marginalized over models according to their posterior probability (see Methods). For each parameter we report the across-tasks compatibility probability *C*_*p*_, that is the (posterior) probability that subjects were best described by the assumption that parameter values were the same across separate tasks, above and beyond chance. The first two rows of parameters compute compatibility across all three tasks, whereas in the last row compatibility only includes the bisensory tasks (bisensory inertial discrimination and unity judgment), as these parameters are irrelevant for the unisensory task.

In sum, our analysis shows that the implicit causal inference data alone are largely inconclusive, possibly because almost all models behave similarly to forced fusion. To further explore our results, we examined the posterior distribution of the prior probability of common cause parameter *p*_c_ across Bayesian models, and of the criterion *κ*_c_ for fixed-criterion models (Fig 5, bottom left panels). In both cases we found a broad distribution of parameters, with only a mild accumulation towards ‘forced fusion’ values (*p*_c_ = 1 or $\kappa_{\text{c}}≳90^{{}^{\circ}}$), suggesting that subjects were not completely performing forced fusion. Thus, it is possible that by constraining the inference with additional data we would be able to draw more defined conclusions.

### Joint model fits

Data from the explicit and implicit causal inference tasks, when analyzed separately, afforded only weak conclusions about subjects’ behavior. The natural next step is to combine datasets from the two tasks along with the data from the unisensory heading discrimination task in order to better constrain the model fits.

Before performing such joint fit, we verified whether there was evidence that model parameters changed substantially across tasks, in which case we might have had to change the structure of the models (e.g., by introducing a subset of distinct parameters for different tasks [49]). For each model parameter, we computed the across-tasks compatibility probability *C*_*p*_ (Fig 5), which is the (posterior) probability that subjects were most likely to have the same parameter values across tasks, as opposed to different parameters, above and beyond chance (see Methods for details). We found at most mild evidence towards difference of parameters across the three tasks, but no strong evidence (all *C*_*p*_ > .05). Therefore, we proceeded in jointly fitting the data with the default assumption that parameters were shared across tasks.

For the joint fits there are nine possible models for the causal inference strategy (three explicit causal inference × three implicit causal inference strategies). However, we considered only a subset of plausible combinations, to avoid ‘model overfitting’ (see Discussion). First, we disregarded the stochastic fusion strategy for the explicit task, since this strategy was strongly rejected by the explicit task data alone. Second, if subjects performed some form of causal inference (Bayesian or fixed-criterion) in both tasks, we forced it to be the same. This reduces the model space for the causal inference strategy to four components: Bay/Bay, Fix/Fix, Bay/FFu, Fix/FFu (explicit/implicit task). Combined with the prior and sensory noise factors as per Fig 2A, this leads to sixteen models.

**Fig 6:**
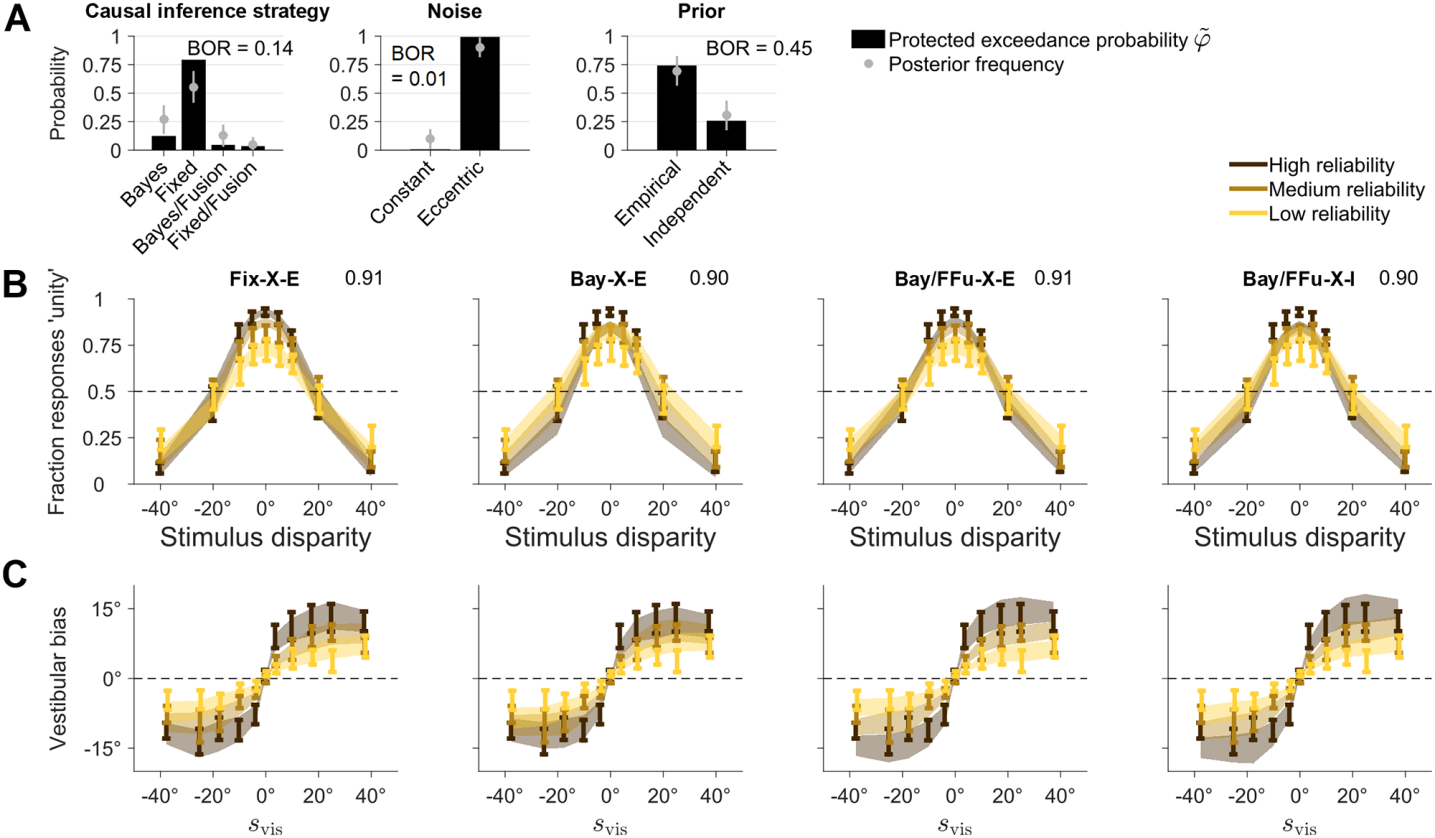
Joint fits. Results of the joint fits across tasks. **A**: Protected exceedance probability $\tilde{\varphi }$ and estimated posterior frequency (mean ± SD) of distinct model components for each model factor. Each factor also displays the Bayesian omnibus risk (BOR). **B**: Joint model fits of the explicit causal inference (unity judgment) task, for different models of interest. Each panel shows the proportion of ‘unity’ responses, as a function of stimulus disparity and for different levels of visual reliability. Bars are ±1 SEM of data across subjects. Shaded areas are ±1 SEM of model predictions across subjects. Numbers on top right of each panel report the absolute goodness of fit across all tasks. **C**: Joint model fits of the implicit causal inference task, for the same models of panel B. Panels show vestibular bias as a function of co-presented visual heading direction *s*_vis_, and for different levels of visual reliability. Bars are ±1 SEM of data across subjects. Shaded areas are ±1 SEM of model predictions across subjects.

Factorial model comparison via LOO found that the most likely causal inference strategy was fixed-criterion ($\tilde{\varphi }=0.79$), followed by Bayesian ($\tilde{\varphi }=0.13$), and then by forced fusion in the implicit task ($\tilde{\varphi }=0.05$ paired with Bayesian explicit causal inference, $\tilde{\varphi }=0.03$ paired with fixed-criterion explicit causal inference; Fig 6A). This is positive evidence that subjects were performing some form of causal inference also in the implicit task, as opposed to mere forced fusion (ratio ∼ 11.4). Moreover, we found strong evidence for eccentricity-dependent over constant noise ($\tilde{\varphi }>0.99$, ratio ∼ 132.7). Instead, the joint data were still inconclusive about the prior adopted by the subjects, with only marginal evidence for the empirical prior over the independent prior (∼ 2.9).

In terms of specific models, the most likely model was fixed-criterion (X-E), followed by Bayesian (X-E), and explicit Bayesian / implicit forced fusion (both X-I and X-E). The best models gave a good description of the individual joint data, with an absolute goodness of fit of ≈ 91% ± 1% (Fig 6B).

Examination of the subjects’ posteriors over parameters for the joint fits (Table 2 and Fig 5, black lines) showed reasonable results. The base visual noise parameters were generally monotonically increasing with decreasing visual cue reliability; the vestibular base noise was roughly of the same magnitude as the medium visual cue noise (as per experiment design); both visual and vestibular noise increased mildly with the distance from straight ahead; subjects had a small lapse probability. For Bayesian models, *p*_c_ was substantially larger than the true value, 0.20 (*t*-test *t*_(10)_ = 10.8, *p* < 10^−4^, *d* = 3.25), suggesting that observers generally thought that heading directions had a higher a priori chance to be the same. Nonetheless, for all but one subject *p*_c_ was far from 1, suggesting that subjects were not performing forced fusion either. An analogous result holds for the fixed criterion *κ*_c_, which was smaller than the largest disparity between heading directions. We found that prior parameters *σ*_prior_ and Δ_prior_ had a lesser impact on the models, and their exact values were less crucial, with generally wide posteriors.

**Table 2:** Joint fit parameters.

Parameter	Description	Posterior mean	Allowed range
All tasks			
*σ*_0__vest_	Vestibular base noise	6.49° ± 0.90°	[0.5°, 80°]†
*σ*_0__vis_(*c*_high_)	Visual base noise (high coherence)	4.08° ± 0.54°	[0.5°, 80°]†
*σ*_0__vis_(*c*_med_)	Visual base noise (medium coherence)	6.32° ± 1.00°	[0.5°, 80°]†
*σ*_0__vis_(*c*_low_)	Visual base noise (low coherence)	11.57° ± 2.67°	[0.5°, 80°]†
*w*_vest_	Vestibular noise eccentricity	0.04 ± 0.01	[0, 1]
*w*_vis_	Visual noise eccentricity	0.07 ± 0.02	[0, 1]
λ	Lapse rate	0.01 ± 0.01	[0, 1]
Bisensory only			
*p*_c_	Prior of common cause (Bay models)	0.56 ± 0.05	[0, 1]
*κ*_c_	Fixed criterion (Fix models)	26.50° ± 3.52°	[0.25°, 180°]†
*σ*_prior_	Central prior width	49.77° ± 12.08°	[1°, 120°]†
Δ_prior_	Disparity prior width	23.51° ± 6.39°	[1°, 120°]†

Posterior means of parameters in the joint fit, marginalized over models according to each subject’s posterior model probability, and averaged across subjects (± SEM). For reference, we also report the parameter range used for the optimization and MCMC sampling.

^†^ These parameters were transformed and fitted in log space.

Finally, we verified that our results did not depend on the chosen comparison metric. Remarkably, the findings regarding causal inference factors were quantitatively the same for all metrics, demonstrating robustness of our main result. Marginal likelihood and BIC differed from LOO and AICc in that they only marginally favored eccentricity-dependent noise models, showing that conclusions over the noise model may depend on the specific choice of metric. All metrics agreed in marginally preferring the empirical prior over the independent prior.

In conclusion, when combining evidence from all available data, our model comparison shows that subjects were most likely performing some form of causal inference instead of forced fusion, for both the explicit and the implicit causal inference tasks. In particular, we find that a fixed-criterion, non-probabilistic decision rule (i.e., one that does not take uncertainty into account) describes the joint data better than the Bayesian strategy, although with some caveats (see Discussion).

### Sensitivity analysis and model validation

Performing a factorial comparison, like any other statistical analysis, requires a number of somewhat arbitrary choices, loosely motivated by previous studies, theoretical considerations, or a preliminary investigation of the data (being aware of the ‘garden of forking paths’ [58]). As good practice, we want to check that our main findings are robust to changes in the setup of the analysis, or be able to report discrepancies.

**Fig 7:**
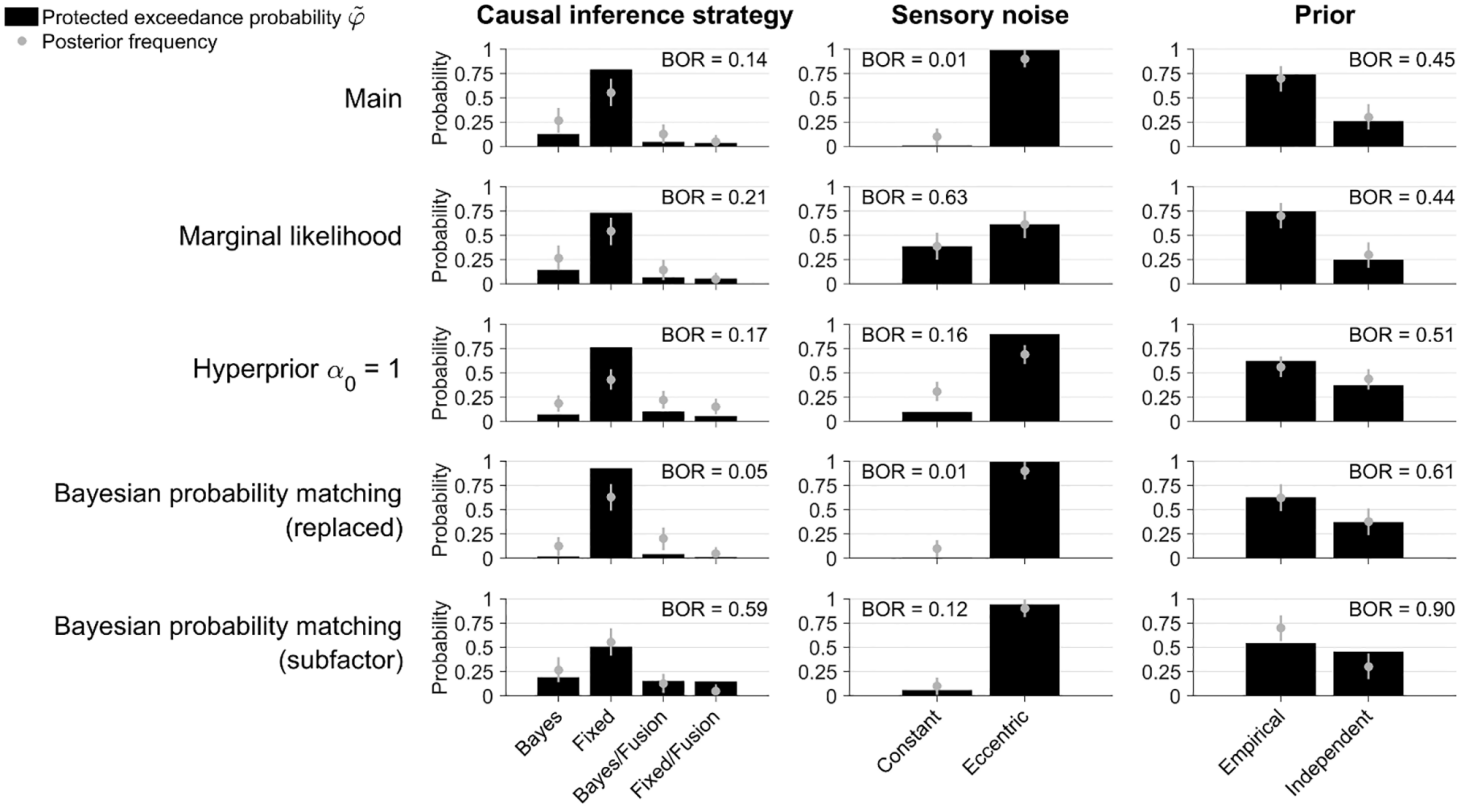
Sensitivity analysis of factorial model comparison. Protected exceedance probability $\tilde{\varphi }$ of distinct model components for each model factor in the joint fits. Each panel also shows the estimated posterior frequency (mean ± SD) of distinct model components, and the Bayesian omnibus risk (BOR). Each row represents a variant of the factorial comparison. 1st row: Main analysis (as per Fig 6A). 2nd row: Uses marginal likelihood as model comparison metric. 3rd row: Uses hyperprior *α*_0_ = 1 for the frequencies over models in the population (instead of a flat prior over model factors). 4th row: Uses ‘probability matching’ strategy for the Bayesian causal inference model (replacing model averaging). 5th row: Includes probability matching as a sub-factor of the Bayesian causal inference family (in addition to model averaging).

We take as our main result the protected exceedance probabilties $\tilde{\varphi }$ of the model factors in the joint analysis (Fig 6A, reproduced in Fig 7, top row). In the following, we examine whether this finding holds up to several manipulations of the analysis framework.

A first check consists of testing different model comparison metrics. In the previous sections, we have reported results for different metrics, finding in general only minor differences from our results obtained with LOO. As an example, we show here the model comparison using as metric an estimate of the marginal likelihood—the probability of the data under the model (Fig 7, 2nd row). We see that the marginal likelihood results agree with our results with LOO except for the sensory noise factor (see Discussion). Therefore, our conclusions about the causal inference strategy are not affected.

Second, the hierarchical Bayesian Model Selection method requires to specify a prior over frequencies of models in the population [54]. This (hyper)prior is specified via the concentration parameter vector ***α***_0_ of a Dirichlet distribution over model frequencies. For our analysis, since we focused on the factorial aspect, we chose an approximately ‘flat’ prior across model factors (see Methods for details), instead of the default flat prior over individual models (*α*_0_ = 1). We found that performing the group analysis with *α*_0_ = 1 did not change our results (Fig 7, 3rd row).

Another potential source of variation is specific model choices, or inclusion of model factors. For example, a common successful variant of the Bayesian causal inference strategy is ‘probability matching’, according to which the observer chooses the causal scenario (*C* = 1 or *C* = 2) randomly, proportionally to its posterior probability [24]. As a first check, we performed the model comparison again using a ‘probability matching’ Bayesian observer *instead* of our main ‘model averaging’ observer (Fig 7, 4th row). Results are similar to the main analysis. If anything, the fixed-criterion causal inference strategy gains additional evidence here, suggesting that probability matching is a worse description of the data than our original Bayesian causal inference model (as confirmed by looking at differences in LOO scores of individual subjects, e.g. for the Bay-X-E model; mean ± SEM: ΔLOO = −17.3 ± 5.7). A recent study in audio-visual causal inference perception has similarly found that probability matching provided a poor explanation of the data [21].

In the factorial framework we could also have performed the previous analysis in a different way, by considering ‘probability matching’ as a sub-factor of the Bayesian strategy, *together* with ‘model averaging’. As we have done before for the explicit causal inference task, we reassign prior probabilities to the models so that they are constant for each factor (in this case, the two Bayesian strategies get a $\times \frac{1}{2}$ multiplier). Results of this alternative approach show an increase of evidence for the Bayesian causal inference family (Fig 7, bottom row). The values of $\tilde{\varphi }$ for the fusion models are also slightly higher, which is due to an increase of the Bayesian omnibus risk (the probability that the observed differences in factor frequencies are due to chance, a warning sign that there are too many models for the available data). This result and other lines of reasoning suggest caution when model factors contain an uneven number of models (see Discussion). Nonetheless, the main conclusion does not qualitatively change, in that observers performed some form of causal inference as opposed to forced fusion.

Finally, we performed several sanity checks, including a model recovery analysis to ensure the integrity of our analysis pipeline and that models of interest were meaningfully distinguishable (see Methods and S1 Appendix for details).

In conclusion, we have shown how the computational framework of Bayesian factorial model comparison, which is made possible by a combination of methods described in the cookbook, allows to explore multiple questions about aspects of subjects’ behavior in multisensory perception, and to account for uncertainty at different levels of the analysis in a principled, robust manner.

## Discussion

We presented a ‘cookbook’ of algorithmic recipes for robust Bayesian evaluation of observer models of causal inference that have widespread applications to multisensory perception and modeling perceptual behavior in general. We applied these techniques to investigate the decision strategies that characterize explicit and implicit causal inference in multisensory heading perception. Examination of observers’ behavior in the explicit and implicit causal inference tasks provided evidence that observers did not simply fuse visual and vestibular cues. Instead, observers integrated the multisensory cues based on their relative disparity, a signature of causal inference. Importantly, our framework affords investigation of whether humans adopt a statistically optimal Bayesian strategy or instead implement a heuristic decision rule which does not fully consider the uncertainty associated with the stimuli.

### Causal inference in multisensory heading perception

Our findings in the explicit causal inference task demonstrate that subjects used information about the discrepancy between the visual and vestibular cues to infer the causal relationship between them. Results in the implicit causal inference task alone were mixed, in that we could not clearly distinguish between alternative strategies, including forced fusion—in agreement with a previous finding [33]. However, when we combined evidence from all tasks, we found that some form of causal inference was more likely than mere forced fusion, in agreement with a more recent study [34]. Our findings suggest that multiple sources of evidence (e.g., different tasks) can help disambiguate causal inference strategies which might otherwise produce similar patterns of behavioral responses.

Our Bayesian analysis allowed us to examine the distribution of model parameters, in particular the causal inference parameters *p*_c_ and *κ*_c_, which govern the tendency to bind or separate cues for, respectively, a Bayesian and a heuristic fixed-criterion strategy. Evidence from all tasks strongly constrained these parameters for each subject. Interestingly, for the Bayesian models we found an average *p*_c_ much higher than the true experimental value (inferred *p*_c_ ∼ 0.5 vs. experimental *p*_c_ = 0.2). This suggests that subjects had a tendency to integrate sensory cues substantially more than what the statistics of the task would require. Note that, instead, a Bayesian observer would be able to learn the correct value of *p*_c_ from noisy observations, provided some knowledge of the structure of the task. Our finding is in agreement with previous studies which demonstrated an increased tendency to combine discrepant visual and vestibular cues [10, 33, 43, 59, 60] and also a large inter-subject variability in *p*_c_, and not obviously related to the statistics of the task [23]. We note that, in all studies so far, the ‘binding tendency’ (*p*_c_ or *κ*_c_) is a descriptive parameter of causal inference models that lacks an independent empirical correlate (as opposed to, for example, noise parameters, which can be independently measured). Understanding the origin of the binding tendency, and which experimental manipulations it is sensitive to, is venue for future work [23, 61]. For example, de Winkel and colleagues found that the binding tendency depends on the duration of the motion stimuli; decreasing for motions of longer duration [34].

Previous work has performed a factorial comparison of only causal inference strategies [21]. Our analysis extends that work by including as latent factors the shape of sensory noise (and, thus, likelihoods) and type of priors [48, 49]. Models in our set include a full computation of the observers’ posterior beliefs based on eccentricity-dependent likelihoods, which was only approximated in previous studies that considered eccentricity-dependence [22, 33, 34]. Indeed, in agreement with a recent finding, we found an important role of eccentricity-dependent noise [22]. Conversely, our analysis of priors was inconclusive, as our datasets were unable to tell whether people learnt the empirical (correlated) prior, or made an assumption of independence.

Our main finding, relative to the causal inference strategy, is that subjects performed causal inference both in the explicit and implicit tasks. Interestingly, from our analyses the most likely causal inference strategy is a fixed-criterion strategy, which crucially differs from the Bayesian strategy in that it does not take cue reliability into account—let alone optimally. This finding is seemingly at odds with a long list of results in multisensory perception, in which people are shown to take cue uncertainty into account [9, 10, 42, 62]. We note that this is not necessarily in contrast with existing literature, for several reasons. First, this result pertains specifically to the causal inference part of the observer model, and not how cues are combined once a common cause has been inferred [21]. To our knowledge, no study of multisensory perception has tested Bayesian models of causal inference against heuristic models that take into account disparity but not reliability, as it has been done for example in visual search [56, 63] and visual categorization [36, 64]. A quantitative modeling approach is needed—qualitatively analyzing the differences in behavior at different levels of reliability is not sufficient to establish that observers take uncertainty into account; patterns of observed differences may be due to a change in sensory noise even if the observer’s decision rule disregards cue reliability. Second, our results are not definitive—the evidence for fixed-criterion vs. Bayesian is positive but not decisive. Our interpretation of this result is that subjects are following some suboptimal decision rule which happens to be closer to fixed-criterion than to the Bayesian strategy for the presented stimuli and range of tested reliability levels. It is possible that with a wider range of stimuli and reliabilities, and possibly with different ways of reporting (e.g., estimation instead of discrimination), we would be able to distinguish the Bayesian strategy from a fixed-criterion heuristic.

Finally, we note that model predictions of our Bayesian models are good but still show systematic discrepancies from the data for the explicit causal inference task (Figs 3C and 6B). Previous work has found similar discrepancies in model fits of unity judgments data across multiple sensory reliabilities (e.g., see Fig 2A in [21]). This suggests that there is some element of model mismatch in current Bayesian causal inference models, possibly due to difference in noise models or to other processes that affect causal inference across cue reliabilities, which deserves further investigation.

### Bayesian factorial comparison

We performed our analysis within a factorial model comparison framework [50]. Even though we were mainly interested in a single factor (causal inference strategy), previous work has shown that the inferred observer’s decision strategy might depend on other aspects of the observer model, such as sensory noise or prior, due to nontrivial interactions of all these model components [37]. Our method, therefore, consisted of performing inference across a family of observer models that explicitly instantiated plausible model variants. We then marginalized over details of specific observer models, looking at posterior probabilities of model factors, according to a hierarchical Bayesian Model Selection approach [54, 55]. We applied a few tweaks to the Bayesian Model Selection method to account for our focus on factors as opposed to individual models (see Methods).

Our approach was fully Bayesian in that we took into account parameter uncertainty (by computing a metric, LOO, based on the full posterior distribution) and model uncertainty (by marginalizing over model components). A fully Bayesian approach has the advantages of explicitly representing uncertainty in the results (e.g., credible intervals over parameters), and of reducing the risk of overfitting, although it is not immune to it [65].

In our case, we marginalized over models to reduce the risk of model overfitting, which is a complementary problem to parameter overfitting. Model overfitting is likely to happen when model selection is performed within a large number of discrete models. In fact, some authors recommend to skip discrete model selection altogether, preferring instead inference and Bayesian parameter estimation in a single overarching or ‘complete’ model [66]. We additionally tried to reduce the risk of model overfitting by balancing prior probabilities across factors, although we noted that this may not be enough to counterbalance the additional flexibility that a model factor gains by having more sub-models than a competitor. Our practical recommendation, until more sophisticated comparison methods are available, is to ensure that all model components within a factor have the same number of models, and to limit the overall number of models.

Our approach was also factorial in the treatment of different tasks, in that first we analyzed each bisensory task in isolation, and then combined trials from all data in a joint fit. The fully Bayesian approach allowed us to compute posterior distributions for the parameters, marginalized over models (see Fig 5), which in turn made it possible to test whether model parameters were compatibile across tasks, via the ‘compatibility probability’ metric. The compatibility probability is an approximation of a full model comparison to test whether a given parameter is the same or should differ across different datasets (in this case, tasks), where we consider ‘sameness’ to be the default (simplyfing) hypothesis. We note that if the identity or not of a parameter across datasets is a main question of the study, its resolution should be addressed via a proper model comparison.

With the joint fits, we found that almost all parameters were well constrained by the data (except possibly for the parameters governing the observers’ priors, *σ*_prior_ and Δ_prior_). An alternative option to better constrain the inference for scarce data or poorly identified parameters is to use informative priors (as opposed to non-informative priors), or a hierarchical approach that assumes a common (hyper)prior to model parameters across subjects [67].

### Model comparison metrics

The general goal of a model comparison metric is to score a model for goodness of fit and somehow penalize for model flexibility. In our analysis we have used Pareto-smoothed importance sampling leave-one-out cross-validation (PSIS-LOO [53]) as the main metric to compare models (simply called LOO in the other sections for simplicity). In fact, there is a large number of commonly used metrics, such as (corrected) Akaike’s information criterion (AIC(c)) [68], Bayesian information criterion (BIC) [68], deviance information criterion (DIC) [69], widely applicable information criterion (WAIC) [70], and marginal likelihood [71]. The literature on model comparison is vast and with different schools of thought—by necessity here we only summarize some remarks. The first broad distinction between these metrics is between predictive metrics (AIC(c), DIC, WAIC, and PSIS-LOO) [72], that try to approximate out-of-sample predictive error (that is, model performance on unseen data), and BIC and marginal likelihood, which try to establish the true model generating the data [71]. Another orthogonal distinction is between metrics based on point estimates (AIC(c) and BIC) vs. metrics that use partial to full information about the model’s uncertainty landscape (DIC, WAIC, PSIS-LOO, based on the posterior, and the marginal likelihood, based on the likelihood integrated over the prior).

First, when computationally feasible we prefer uncertainty-based metrics to point estimates, since the latter are only crude asymptotic approximations that do not take the model and the data into account, besides simple summary statistics (number of free parameters and possibly number of data points). Due to their lack of knowledge of the actual structure of the model, AIC(c) and BIC can grossly misestimate model complexity [72].

Second, we have an ordered preference among predictive metrics, that is PSIS-LOO ≻ WAIC ≻ DIC ≻ AIC(c) [72]. The reason is that all of these metrics more or less asymptotically approximate full leave-one-out cross validation, with increasing degree of accuracy from right to left [53, 72]. As mentioned before, AIC(c) works only in the regime of a large amount of data. DIC, albeit commonly used, has several issues and requires the posterior to be multivariate normal, or at least symmetric and unimodal—gross failures can happen when this is not the case, since DIC bases its estimate of model complexity on the mean (or some other measure of central tendency) of the posterior [72]. WAIC is a great improvement over DIC and does not require normality of the posterior, but its approximation is generally superseded by PSIS-LOO [53]. Moreover, PSIS-LOO has a natural diagnostic, the exponents of the tails of the fitted Pareto distribution, which allows the user to know when the method may be in trouble [53]. Full leave-one-out cross validation is extremely expensive, but PSIS-LOO only requires the user to compute the posterior via MCMC sampling, with no additional cost with respect to DIC or WAIC. Similarly to WAIC, PSIS-LOO requires the user to store for each posterior sample the log likelihood *per trial*, which with modern computers represent a negligible storage cost.

The marginal likelihood, or Bayes factor (of which BIC is a poor approximation), is an alternative approach to quantify model evidence, related to computing the posterior probability of the models [71]. While this is a principled approach, it entails several practical and theoretical issues. First, the marginal likelihood is generally hard to compute, since it usually involves a complicated, high-dimensional integral of the likelihood over the prior (although this computation can be simplified for nested models [73]). Here, we have applied a novel approximation method for the marginal likelihood following ideas delineated in [74, 75], obtaining generally sensible values. However, more work is needed to establish the precision and applicability of such technique. Besides practical computational issues, the marginal likelihood, unlike other metrics, is sensitive to the choice of prior over parameters, in particular its range [66]. Crucially, and against common intuition, this sensitivity does not reduce with increasing amounts of data. A badly chosen (e.g., excessively wide) prior for a non-shared parameter might change the marginal likelihood of a model by several points, thus affecting model ranking. The open issue of prior sensitivity has led some authors to largely discard model selection based on the marginal likelihood [66].

For these reasons, we chose (PSIS-)LOO as the main model comparison metric. As a test of robustness, we also computed other metrics and verified that our results were largely independent of the chosen metric, or investigated the reasons when it was not the case.

As a specific example, in our analysis we found that LOO and marginal likelihood (or BIC) generally agreed on all comparisons, except for the sensory noise factor. Unlike LOO, the marginal likelihood tended to prefer constant noise models as opposed to eccentricity-dependent models. Our explanation of this discrepancy is that for our tasks eccentricity-dependence provides a consistent but small improvement to the goodness of fit of the models, which can be overrided by a large penalty due to model complexity (BIC), or to the chosen prior over the eccentricity-dependent parameters (*w*_vis_, *w*_vest_), whose range was possibly wider than needed (see Fig 5). The issue of prior sensitivity (specifically, dependence of results on an arbitrarily chosen range) can be attenuated by adopting a Bayesian hierarchical approach over parameters (or a more computationally feasibile approximation, known as empirical Bayes), which is venue for future work.

### Computational framework

Model evaluation, especially from a Bayesian perspective, is a time-consuming business. For this reason, we have compiled several state-of-the-art methods for model building, fitting and comparison, and made our code available.

The main issue of many common observer models in perception is that the expression for the (log) likelihood is not analytical, requiring numerical integration or simulation. To date, this limits the applicability of modern model specification and analysis tools, such as probabilistic programming languages, that exploit auto-differentiation and gradient-based sampling methods (e.g., Stan [76] or PyMC3 [77]). The goal of such computational frameworks is to remove the burden and technical details of evaluating the models from the shoulders of the modeler, who only needs to provide a model specification.

In our case, we strive towards a more modest goal of providing black-box algorithms for optimization and MCMC sampling that exhibit a larger degree of robustness than standard methods. In particular, for optimization (maximum likelihood estimation) we recommend Bayesian adaptive direct search (BADS [78]), a technique based on Bayesian optimization [79, 80], which exhibits robustness to noise and jagged likelihood landscapes, unlike common optimization methods such as fminsearch (Nelder-Mead) and fmincon in MATLAB. Similarly, for MCMC sampling we propose a sampling method that combines the robustness and self-adaptation of slice sampling [81] and ensemble-based methods [82]. Crucially, our proposed method almost completely removes the need of expensive trial-and-error tuning on the part of the modeler, possibly one of the main reasons why MCMC methods and full evaluation of the posterior are relatively uncommon in the field (to our knowledge, this is the first study of causal inference in multisensory perception to adopt a fully Bayesian approach).

Our framework is similar to the concept behind the VBA toolbox, a MATLAB toolbox for probabilistic treatment of nonlinear models for neurobiological and behavioral data [83]. The VBA toolbox tackles the problem of model fitting via a variational approximation that assumes factorized, Gaussian posteriors over the parameters (mean field/Laplace approximation), and provides the variational free energy as an approximation (lower bound) of the marginal likelihood. Our approach, instead, does not make any strong assumption, using MCMC to recover the full shape of the posterior, and state-of-the-art techniques to assess model performance.

Detailed, rigorous modeling of behavior is a necessary step to constrain the search for neural mechanisms implementing decision strategies [84] We have provided a set of computational tools and demonstrated how they can be applied to answer specific questions about internal representation and decision strategies of the observer in multisensory perception, with the goal of increasing the set of models that can be investigated, and the robustness of such analyses. Thus, our tools can be of profound use not only to the field of multisensory perception, but to biological modeling in general.

## Methods

### Ethics statement

The Institutional Review Board at the Baylor College of Medicine approved the experimental procedures (protocol number H-29411, “Psychophysics of spatial orientation and vestibular influences on spatial constancy and movement planning”) and all subjects gave written informed consent.

### Human psychophysics

#### Subjects

Eleven healthy adults (4 female; age 26.4 ± 4.6 years, mean ± SD) participated in the full study. Subjects had no previous history of neurological disorders and had normal or corrected-to-normal vision. Four other subjects completed only a partial version of the experiment, and their data were not analyzed here.

#### Apparatus

Details of the experimental apparatus have been previously published and are only described here briefly [9, 14, 85, 86]. Subjects were seated comfortably in a cockpit-style chair and were protectively restrained with a 5-point racing safety harness. Each subject wore a custom-made thermoplastic mesh mask that was attached to the back of the chair for head stabilization. The chair, a three-chip DLP projector (Galaxy 6; Barco) and a large projection screen (149 × 127 cm) were all mounted on a motion platform (6DOF2000E; Moog, Inc.). The projection screen was located ∼65 cm in front of the eyes, subtending a visual angle of ∼94° × 84°. Subjects wore LCD-based active 3D stereo shutter glasses (Crystal Eyes 4, RealD, Beverly Hills) to provide stereoscopic depth cues and headphones for providing trial timing-related feedback (a tone to indicate when a trial was about the begin and another when a button press was registered). This apparatus was capable of providing three self-motion conditions: vestibular (inertial motion through the movement of the platform), visual (optic flow simulating movement of the observer in a 3D virtual cloud of stars, platform stationary) and combined visual-vestibular heading (temporally-synchronized optic flow and platform motion) at various spatial discrepancies.

#### Stimuli

We modified a previous multisensory heading discrimination task [9]. Here subjects experienced combined visual and vestibular translation in the horizontal plane (Fig 1A). The visual scene and platform movement followed a Gaussian velocity profile (displacement = 13 cm, peak Gaussian velocity = 26 cm/s and peak acceleration = 0.9m/s^2^, duration = 1 s). Visual and vestibular headings were either in the same direction or their movement trajectories were separated by a directional disparity, Δ, expressed in degrees (Fig 1A). The directional disparity Δ and visual cue reliability were varied on a trial-by-trial basis. Δ took one of five values, selected with equal probability: 0° (no conflict), 5°, 10°, 20° and 40°. Thus, visual and vestibular stimuli were in conflict in 80% of the trials. In each trial, Δ was randomly assigned to be positive (Fig 1A right, vestibular heading to the right of visual heading) or negative. Once a disparity value, Δ, was chosen, the mean heading angle ($\bar{s}$) which represents the average of vestibular and visual headings, was uniformly randomly drawn from the discrete set {−25°, −20°, …, 25°}. Vestibular heading (*s*_vest_, red trace in Fig 1) and visual heading (*s*_vis_, black trace in Fig 1A) were generated by displacing the platform motion and optic flow on either side of the mean heading by Δ/2. The vestibular and visual headings experienced by subjects were defined as $s_{\text{vest}}=\bar{s}+\Delta /2$ and $s_{\text{vis}}=\bar{s}-\Delta /2$, respectively. This procedure entailed that visual and vestibular heading directions presented in experiment were correlated (Fig 1B). Three levels of visual cue reliability (high, medium, and low) were tested. Visual reliability was manipulated by varying the percentage of stars in the optic flow that coherently moved in the specified heading direction. For all subjects, visual motion coherence at high reliability was set at 100%. Coherence at medium reliability was selected for each subject during a preliminary session via a manual staircasing procedure such that their visual and vestibular thresholds were approximately matched. Coherence at low reliability was also selected for each subject separately and this was a value that was chosen to be lower than the medium reliability. Thus, the optic flow coherences for medium and low reliabilities were different across subjects with ranges of 40-70% and 25-50%, respectively. Overall, there were 297 stimulus conditions (9 directional disparities × 11 mean heading directions × 3 visual cue reliabilities) which were randomly interleaved.

#### Tasks

First, subjects (*n* = 11) performed in a session of a unisensory heading discrimination task (left/right of straight ahead), in which visual or vestibular stimuli were presented in isolation. Vestibular stimuli had one fixed reliability level, whereas visual stimuli were tested on three different reliability levels, randomly interleaved, resulting in a total of 350–750 trials.

Then, subjects performed two-three sessions of the explicit causal inference task (unity judgment). Here, subjects indicated if the visual and vestibular cues indicated heading in the same direction (“common” cause, *C* = 1) or in different directions (“different” causes, *C* = 2). Each combination of disparity and reliability was presented at least 20 times. Since each disparity was randomly assigned to be positive or negative on each trial, 0° disparity was presented at least 40 times at each visual cue reliability resulting in a total of 700-1200 trials. Subjects did not receive feedback about the correctness of their responses.

Finally, the same subjects also participated in the implicit causal inference task—bisensory (inertial) discrimination. Here, subjects indicated the perceived direction of their inertial self-motion (left or right of straight ahead). Note that although both visual and vestibular stimuli were presented in each trial, subjects were asked to only indicate their perceived direction of inertial heading, similar to the bisensory auditory localization procedure in [21]. Each combination of disparity and visual cue reliability was presented at least 70 times. Since each disparity was randomly assigned to be positive or negative on each trial, 0° disparity was presented at least 140 times resulting in a total of 2100-3000 trials divided across 7-9 sessions. No feedback was given about the correctness of subjects’ responses.

For all tasks, sessions were about one hour long and subjects were required to take multiple breaks within each session.

#### Data analysis

For the explicit causal inference task, we computed the proportion of trials in which subjects perceived a common cause at each disparity and visual cue reliability. For the implicit causal inference task, we calculated the shift in perceived inertial heading as a function of *s*_vis_, that is the influence that *s*_vis_ had on *s*_vest_, and we called this model-free summary statistic ‘bias’. In order to build psychometric functions with enough trials, we binned values of *s*_vis_ in the following intervals: {[−45°, −30°], [−27.5°, −22.5°], [−20°, −15°], [−12.5°, −7.5°], [−5°, −2.5°], 0°, [2.5°, 5°], [7.5°, 12.5°], [15°, 20°], [22.5°, 27.5°], [30°, 45°]}. Bin ranges were chosen to yield a comparable number of trials per bin, according to the nonuniform distribution of *s*_vis_ in the experiment (see Fig 1B). For each visual bin and level of visual cue reliability, we constructed psychometric functions by fitting the proportion of rightward responses as a function of *s*_vest_ with cumulative Gaussian functions (inset in Fig 3A). Thus, we defined the bias in the perceived inertial heading as minus the point of subjective equality (L/R PSE). A bias close to zero indicates that subjects accurately perceived their inertial (vestibular) heading. Large shifts of the PSE away from zero, that is substantial biases, suggest that misleading visual cues exerted a significant influence on the accuracy of inertial heading discrimination. Note that we do not expect the psychometric curves to be *exact* cumulative Gaussian functions, because of nonlinearities due to eccentricity-dependence of the noise and effects of causal inference. Nonetheless, the bias as we defined it is useful as a simple model-free statistic. Repeated-measures ANOVA with disparity or visual bin and visual cue reliability as within-subjects factors were performed separately on the proportion of common cause reports and bias in perceived inertial heading. We applied Greenhouse-Geisser correction of the degrees of freedom in order to account for deviations from sphericity [87], and report effect sizes as partial eta squared, denoted with $\eta_{p}^{2}$. For all analyses the criterion for statistical significance was *p* < .05, and we report uncorrected *p*-values. Unless specified otherwise, summary statistics are reported in the text as mean ± SE between subjects. Finally, we remark that the summary statistics described above were used only for visualization and to perform simple descriptive statistics; we fit all models to raw trial data.

### Causal inference models

We build upon standard causal inference models of multisensory perception [18]. For concreteness, in the following description of causal inference models we refer to the visuo-vestibular example with binary responses (‘left/right’ for discrimination, and ‘yes/no’ for unity judgements). The basic component of any observer model is the trial response probability, that is the probability of observing a given response for a given trial condition (e.g., stimulus pair, uncertainty level, task). In the following we briefly review how these probabilities are computed.

All analysis code was written in MATLAB (Mathworks, Inc.), with core computations in C for increased performance (via *mex* files in MATLAB). Code is available at https://github.com/lacerbi/visvest-causinf.

#### Unisensory heading discrimination

We used subjects’ binary (‘left or right of straight forward’) heading choices, measured in the presence of visual-only and vestibular-only stimuli, to estimate subjects’ measurement noise in the respective sensory signals. Let us consider a trial with a vestibular-only stimulus (the computation for a visual-only stimulus is analogous). Subjects are asked whether the perceived direction of motion *s*_vest_ is to the left or to the right of straight forward (0°). We assume that the observer has access to a noisy measurement *x*_vest_ of stimulus *s*_vest_ (direction of motion), with probability density

$$\begin{array}{c} p\left(x_{\text{vest}}|s_{\text{vest}}\right)=N(x_{\text{vest}}|s_{\text{vest}},\sigma^{2}\left(s_{\text{vest}}\right)), \end{array}$$ (1)

where $N(x|\mu ,\sigma^{2})$ is a normal probability density with mean *μ* and variance *σ*^2^. Since stimulus directions are defined over the circle, we also considered a wrapped normal or, similarly, a von Mises distribution instead of Eq 1. Because of the relatively small range of stimuli used in the experiment, we found no difference between the distributions defined over the full circle and the simple normal distribution in Eq 1 (see S1 Appendix). Incidentally, in an additional investigation we also found no empirical difference between a wrapped normal and a von Mises, so either noise distribution could be used in the presence of fully circular stimuli (see S1 Appendix).

Depending on the sensory noise model, the variance in Eq 1 is either *constant* ($\sigma^{2}\left(s_{\text{vest}}\right)\equiv {\sigma_{0}^{2}}_{\text{vest}}$) or *eccentricity-dependent* with base magnitude ${\sigma_{0}^{2}}_{\text{vest}}$ and noise that increases with eccentricity (distance from 0°) approximately quadratically, at least for small headings, according to a parameter *w*_vest_ ≥ 0 (see S1 Appendix for details). For *w*_vest_ = 0, the eccentricity-dependent model reduces to the constant model. The observer’s posterior probability density over the vestibular stimulus is *p*(*s*_vest_|*x*_vest_) ∝ *p*(*x*_vest_|*s*_vest_)*p*_prior_(*s*_vest_), and we will see that under some assumptions the prior over heading directions is irrelevant for subsequent computations in the left/right unisensory task (see S1 Appendix).

We assume that observers compute the posterior probability that the stimulus is right of straight forward as $\text{Pr}\left(s_{\text{vest}}>0|x_{\text{vest}}\right)=\int_{0}^{90}p\left(s_{\text{vest}}|x_{\text{vest}}\right)ds_{\text{vest}}$, and respond ‘right’ if Pr(*s*_vest_ > 0|*x*_vest_) > 0.5; ‘left’ otherwise (see S1 Appendix for details). Observers may also lapse and give a completely random response with probability λ (lapse rate). This yields

$$\begin{array}{c} \text{Pr}\left(\text{choose}\;\text{right}|x_{\text{vest}}\right)=\frac{\lambda }{2}+(1-\lambda )⟦\;\text{Pr}\left(s_{\text{vest}}>0|x_{\text{vest}}\right)>0.5⟧, \end{array}$$ (2)

where ⟦·⟧ is Iverson bracket, which is 1 if the argument is true, and 0 otherwise [88].

An analogous derivation is applied to each unisensory visual stimulus condition for respectively low, medium, and high visual reliability. We assume a distinct *σ*_0__vis_ for each visual reliability condition, and, for the eccentricity-dependent models, a common *w*_vis_ for all visual reliability conditions, so as to reduce model complexity.

#### Unity judgment (explicit causal inference)

In a unity judgment trial, the observer explicitly evaluates whether there is a single cause (*C* = 1) underlying the noisy measurements *x*_vis_, *x*_vest_, or two separate causes (*C* = 2; see Fig 2B). All following probability densities are conditioned on *c*_vis_, the level of visual cue reliability in the trial, which is assumed to be known to the observer; we omit this dependence to reduce clutter. We consider three families of explicit causal inference strategies.

The *Bayesian* causal inference strategy computes the posterior probability of common cause

$$\begin{array}{c} \text{Pr}\left(C=1|x_{\text{vis}},x_{\text{vest}}\right)=\frac{p\left(x_{\text{vis}},x_{\text{vest}}|C=1\right)p_{\text{c}}}{p\left(x_{\text{vis}},x_{\text{vest}}|C=1\right)p_{\text{c}}+p\left(x_{\text{vis}},x_{\text{vest}}|C=2\right)(1-p_{\text{c}})}, \end{array}$$ (3)

where 0 ≤ *p*_c_ ≡ Pr(*C* = 1) ≤ 1, the prior probability of a common cause, is a free parameter of the model. The derivation of *p*(*x*_vis_, *x*_vest_|*C* = *k*), for *k* = 1, 2, is available in S1 Appendix. The observer reports unity if the posterior probability of common cause is greater than 0.5, with the added possibility of random lapse,

$$\begin{array}{c} \begin{array}{c} \text{Pr}(\text{choose}\;\text{unity}|x_{\text{vis}},x_{\text{vest}})=\frac{\lambda }{2}+\left(1-\lambda \right)⟦\;\text{Pr}\left(C=1|x_{\text{vis}},x_{\text{vest}}\right)>0.5⟧. \end{array} \end{array}$$ (4)

For a separate analysis we also considered a ‘probability matching’ variant that reports unity with probability equal to Pr(*C* = 1|*x*_vis_, *x*_vest_) (plus lapses).

As a non-Bayesian causal inference heuristic model, we consider a *fixed criterion* observer, who reports a common cause whenever the two noisy measurements are within a distance *κ*_c_ ≥ 0 from each other,

$$\begin{array}{c} \begin{array}{c} \text{Pr}(\text{choose}\;\text{unity}|x_{\text{vis}},x_{\text{vest}})=\frac{\lambda }{2}+\left(1-\lambda \right)⟦\;|x_{\text{vis}}-x_{\text{vest}}|<\kappa_{\text{c}}⟧. \end{array} \end{array}$$ (5)

Crucially, the fixed criterion observer does not take into account stimulus reliability or other statistical information when inferring the causal structure.

Finally, we consider a *fusion* observer that eschews causal inference altogether. A classical ‘forced fusion’ observer would *always* report ‘unity’ in the explicit causal inference task, which is easily rejected by the data. Instead, we consider a *stochastic fusion* observer that reports ‘unity’ with probability *η*_low_, *η*_med_, or *η*_high_, depending only on the reliability of the visual cue, and discards any other information.

#### Bisensory inertial discrimination (implicit causal inference)

In bisensory inertial discrimination trials, the observer reports whether the perceived inertial heading *s*_vest_ is to the left or right of straight forward (0°). In this experiment, we do not ask subjects to report *s*_vis_, but the inference would be analogous. The inertial discrimination task requires an implicit evaluation of whether there is a single cause to the noisy measurements *x*_vis_, *x*_vest_ (*C* = 1), or two separate causes (*C* = 2), for a known level of visual coherence *c*_vis_ (omitted from the notation for clarity).

If the observer knew that *C* = *k*, for *k* = 1, 2, the posterior probability density over the vestibular stimulus would be (see S1 Appendix)

$$p(s_{\text{vest}}|x_{\text{vis}},x_{\text{vest}},C=k)\propto \int_{-90^{{}^{\circ}}}^{90^{{}^{\circ}}}p\left(x_{\text{vest}}|s_{\text{vest}}\right)p\left(x_{\text{vis}}|s_{\text{vis}},c_{\text{vis}}\right)p\left(s_{\text{vis}},s_{\text{vest}}|C=k\right)ds_{\text{vis}},$$

where the likelihoods are defined as per the uni-sensory task, Eq 1, and for the prior over heading directions, *p*(*s*_vis_, *s*_vest_|*C*), see ‘Observers’ priors’ below.

The posterior probability of rightward motion is computed for *k* = 1, 2 as

$$\begin{array}{cc} & \text{Pr}\left(s_{\text{vest}}>0|x_{\text{vest}},x_{\text{vis}},C=k\right)\propto \int_{0^{{}^{\circ}}}^{90^{{}^{\circ}}}p(s_{\text{vest}}|x_{\text{vis}},x_{\text{vest}},C=k)ds_{\text{vest}}, \end{array}$$

and an analogous equation holds for the posterior probability of leftward motion.

In general, the causal structure is implicitly inferred by the observer. We assume that observers combine cues according to

$$\begin{array}{c} \begin{array}{cc} p(s_{\text{vest}}|x_{\text{vis}},x_{\text{vest}})= & v_{1}\left(x_{\text{vis}},x_{\text{vest}}\right)\cdot p\left(s_{\text{vest}}|x_{\text{vis}},x_{\text{vest}},C=1\right)+\\ & [1-v_{1}\left(x_{\text{vis}},x_{\text{vest}}\right)]\cdot p\left(s_{\text{vest}}|x_{\text{vis}},x_{\text{vest}},C=2\right) \end{array} \end{array}$$ (6)

where 0 ≤ *v*_1_(*x*_vis_, *x*_vest_) ≤ 1 is the *implicit causal weight* associated by the observer to the hypothesis of a single cause, *C* = 1. The form of the causal weight depends on the observer’s implicit causal inference strategy.

We consider three families of implicit causal inference. For the *Bayesian* causal inference observer, the causal weight is equal to the posterior probability, *v*_1_(*x*_vis_, *x*_vest_) = Pr(*C* = 1|*x*_vis_, *x*_vest_), so that Eq 6 becomes the expression for Bayesian model averaging [18] (see Eq 3 and S1 Appendix). As a variant of the Bayesian observer we consider a *probability matching* Bayesian strategy for which *v*_1_ = 1 with probability Pr(*C* = 1|*x*_vis_, *x*_vest_), and *v*_1_ = 0 otherwise. For the *fixed-criterion* observer, *v*_1_ = ⟦|*x*_vis_ − *x*_vest_| < *κ*_c_⟧, with *κ*_c_ ≥ 0 as per Eq 5. Finally, for the *forced fusion* observer *v*_1_ ≡ 1.

The posterior probability of rightward motion is then $\text{Pr}\left(s_{\text{vest}}>0|x_{\text{vest}},x_{\text{vis}}\right)=\int_{0^{{}^{\circ}}}^{90^{{}^{\circ}}}p\left(s_{\text{vest}}|x_{\text{vis}},x_{\text{vest}}\right)ds_{\text{vest}}$, and an analogous equation holds for the posterior probability of leftward motion. We assume the observer reports the direction with highest posterior probability, with occasional lapses (see also Eq 2),

$$\begin{array}{c} \text{Pr}\left(\text{choose}\;\text{right}|x_{\text{vis}},x_{\text{vest}}\right)=\frac{\lambda }{2}+(1-\lambda )⟦\text{Pr}\left(s_{\text{vest}}>0|x_{\text{vis}},x_{\text{vest}}\right)>0.5⟧, \end{array}$$ (7)

where λ ≥ 0 is the lapse rate.

#### Observers’ prior

We assume subjects develop a symmetric, unimodal prior over heading directions for unisensory trials. Due to the form of the decision rule (Eq 2), a symmetric prior has no effect on the unisensory trials, so we only focus on the bisensory case.

For the bisensory prior over heading directions, *p*(*s*_vis_, *s*_vest_|*C*) we consider two families of priors. The *empirical* prior approximately follows the correlated structure of the discrete distribution of vestibular and visual headings presented in the experiment (Fig 1B). The *independent* prior assumes that observers learn a generic uncorrelated Gaussian prior over heading directions, as per [18]. See S1 Appendix for details.

We note that previous work in heading perception has found a ‘repulsive’ bias away from straight ahead [89, 90], which is seemingly at odds with the central prior assumed here. However, the repulsion bias previously reported can be explained by the current Bayesian framework by means of a stimulus-dependent likelihood [91, 92]. According to the Bayesian theory, such a stimulus-dependent likelihood may induce a bias away from regions of higher sensory precision. Whether the net bias is going to be attractive or repulsive depends on the relative contribution of prior and likelihood [93]. Thus, our models that combine a central prior and stimulus-dependent likelihood are not incompatible with previous findings of repulsive biases. See also S1 Appendix.

#### Trial response probabilities

Eqs 2, 4, 5 and 7 represent the probability that an observer chooses a specific response *r* (‘rightward’ or ‘leftward’ for discrimination trials, ‘same’ or ‘different’ for unity judgment trials), for given noisy measurements *x*_vis_ and *x*_vest_ (or only one of the two for the unisensory task), and known visual reliability *c*_vis_. Since as experimenters we do not have access to subjects’ internal measurements, to compute the trial response probabilities we integrate (‘marginalize’) over the unseen noisy measurements for given heading directions *s*_vis_ and *s*_vest_ presented in the trial.

For the unisensory case, considering as example the vestibular case, we get

$$\begin{array}{c} \text{Pr}\left(\text{observed}\;r|s_{\text{vest}}\right)=\int_{-90^{{}^{\circ}}}^{90^{{}^{\circ}}}\text{Pr}(\text{choose}\;r|x_{\text{vest}})p\left(x_{\text{vest}}|s_{\text{vest}}\right)dx_{\text{vest}}. \end{array}$$ (8)

For the bisensory case, either unity judgment or inertial discrimination, we have

$$\begin{array}{c} \begin{array}{cc} \text{Pr}(\text{observed}\;r|s_{\text{vis}},s_{\text{vest}},c_{\text{vis}})= & \int_{-90^{{}^{\circ}}}^{90^{{}^{\circ}}}\int_{-90^{{}^{\circ}}}^{90^{{}^{\circ}}}\text{Pr}(\text{choose}\;r|x_{\text{vis}},x_{\text{vest}},c_{\text{vis}})\\ & \times p\left(x_{\text{vest}}|s_{\text{vest}}\right)p\left(x_{\text{vis}}|s_{\text{vis}},c_{\text{vis}}\right)dx_{\text{vest}}dx_{\text{vis}}. \end{array} \end{array}$$ (9)

It is customary in the causal inference literature to approximate these integrals via Monte Carlo sampling, by drawing a large number of noisy measurements from the noise distributions (e.g., [18, 20, 24, 33]). Instead, we computed the integrals via numerical integration, which is more efficient than Monte Carlo techniques for low dimensional problems [94]. We used the same numerical approach to evaluate Eqs 2, 4, 5 and 7, including an adaptive method for choice of integration grid. All numerical integrals were then coded in C (*mex* files in MATLAB) for additional speed. See S1 Appendix for computational details.

### Model fitting

For a given model, we denote its set of parameters by a vector ***θ***. For a given model and dataset, we define the parameter log likelihood function as

$$\begin{array}{c} \begin{array}{cc} \text{LL}(\theta ,\text{model}) & =\log p(\text{data}|\theta ,\text{model})\\ & =\log\prod_{i=1}^{N_{\text{trials}}}p(r^{\left(i\right)}|s_{\text{vis}}^{\left(i\right)},s_{\text{vest}}^{\left(i\right)},c_{\text{vis}}^{\left(i\right)},\theta ,\text{model})\\ & =\sum_{i=1}^{N_{\text{trials}}}\log p(r^{\left(i\right)}|s_{\text{vis}}^{\left(i\right)},s_{\text{vest}}^{\left(i\right)},c_{\text{vis}}^{\left(i\right)},\theta ,\text{model}) \end{array} \end{array}$$ (10)

where we assumed conditional independence between trials; *r*^(*i*)^ denotes the subject’s response (‘right’ or ‘left’ for the discrimination trials; ‘common’ or ‘separate’ causes in unity judgment trials); $s_{\text{vis}}^{\left(i\right)}$ and $s_{\text{vest}}^{\left(i\right)}$ are, respectively, the direction of motion of the visual (resp. vestibular) stimulus (if present), and $c_{\text{vis}}^{\left(i\right)}$ is the visual coherence level (that is, reliability: low, medium, or high), in the *i*-th trial.

#### Maximum likelihood estimation

First, we fitted our models to the data via maximum likelihood estimation, by finding the parameter vector θ* that maximizes the log likelihood in Eq 10. For optimization of the log likelihood, we used Bayesian Adaptive Direct Search (BADS; https://github.com/lacerbi/bads). BADS is a black-box optimization algorithm that combines a mesh-adaptive direct search strategy [95] with a local Bayesian optimization search step based on Gaussian process surrogates (see [80, 96] for an introduction to Bayesian optimization). Bayesian optimization is particularly useful when the target function is costly to evaluate or the likelihood landscape is rough, as it is less likely to get stuck in local optima than other algorithms, and may reduce the number of function evaluations to find the (possibly global) optimum. In our case, evaluation of the log likelihood function for a single parameter vector ***θ*** could take up to ∼ 2-3 s for bisensory datasets, which makes it a good target for Bayesian optimization. We demonstrated in a separate benchmark that BADS is more effective than a large number of other MATLAB optimizers for our problem (‘causal inference’ problem set in [78]). See S1 Appendix for more details about the algorithm and the optimization procedure.

For each subject we first fitted separately the datasets corresponding to three tasks (unisensory and bisensory heading discrimination, unity judgment), and then performed joint fits by combining datasets from all tasks (summing the respective log likelihoods).

#### Posterior sampling

As a complementary approach to ML parameter estimation, for each dataset and model we calculated the posterior distribution of the parameters,

$$\begin{array}{c} p(\theta |\text{data},\text{model})\propto p(\text{data}|\theta ,\text{model})p(\theta |\text{model}), \end{array}$$ (11)

where *p*(data|***θ***, model) is the likelihood (see Eq 10) and *p*(***θ***|model) is the prior over parameters. We assumed a factorized prior $p\left(\theta |\text{model}\right)=\prod_{i=1}^{k}p\left(\theta_{i}\right)$ and a non-informative uniform prior over a bounded interval for each model parameter (uniform in log space for scale parameters such as all noise base magnitudes, fixed criterion *κ*_c_, and prior parameters *σ*_prior_ and Δ_prior_); see Table 2.

We approximated Eq 11 via Markov Chain Monte Carlo (MCMC) sampling. We used a custom-written sampling algorithm that combines slice sampling [81] with adaptive direction sampling [82] and a number of other tricks (https://github.com/lacerbi/eissample). Slice sampling is a flexible MCMC method that, in contrast with the common Metropolis-Hastings transition operator, requires very little tuning in the choice of length scale. Adaptive direction sampling is an ensemble MCMC method that shares information between several dependent chains (also called ‘walkers’ [97]) in order to speed up mixing and exploration of the state space. For details about the MCMC algorithm and the sampling procedure, see S1 Appendix.

### Factorial model comparison

We built different observer models by factorially combining three factors: causal inference strategy (Bayesian, fixed-criterion, or fusion); shape of sensory noise (constant or eccentricity-dependent); and type of prior over heading directions (empirical or independent); see Fig 2A and ‘Causal inference models’ section of the Methods for a description of the different factors.

For each subject, we fitted the different observer models, first separately to different tasks (unity judgment and bisensory inertial discrimination), and then performed a joint fit by combining datasets from all tasks (including the unisensory discrimination task). We evaluated the fits with a number of model comparison metrics and via an objective goodness of fit metric. Finally, we combined evidence for different model factors across subjects with a hierarchical Bayesian approach.

We verified our ability to distinguish different models with a model recovery analysis, described in S1 Appendix.

#### Model comparison metrics

For each dataset and model we computed a number of different model comparison metrics, all of which take into account quality of fit and penalize model flexibility, but with different underlying assumptions.

Based on the maximum likelihood solution, we computed Akaike information criterion with a correction for sample size (AICc) and Schwarz’s ‘Bayesian’ Information criterion (BIC),

$$\begin{array}{c} \begin{array}{cc} \text{AICc}= & -2LL\left(\theta^{*}\right)+2k+\frac{2k(k+1)}{N_{\text{trials}}-k-1}\\ \text{BIC}= & -2LL\left(\theta^{*}\right)+k\log N_{\text{trials}} \end{array} \end{array}$$ (12)

where *N*_trials_ is the number of trials in the dataset and *k* is the number of parameters of the model. The factor of −2 that appears in both definitions is due to historical reasons, so that both metrics have the same scale of the deviance.

To assess model performance on unseen data, we performed Bayesian leave-one-out (LOO) cross-validation. Bayesian LOO cross-validation computes the posterior of the parameters given *N*_trials_ − 1 trials (training), and evaluates the (log) expected likelihood of the left-out trial (test); the procedure is repeated for each trial, yielding the leave-one-out score

$$\begin{array}{c} \text{LOO}=\sum_{i=1}^{N_{\text{trials}}}\log\int p(r_{i}|\theta )p\left(\theta |D_{-i}\right)d\theta , \end{array}$$ (13)

where *p*(*r*_*i*_|***θ***) is the likelihood associated to the *i*-th trial alone, and $p(\theta |D_{-i})$ is the posterior over ***θ*** given all trials except the *i*-th one. Eq 13 can be estimated at prohibitive computational cost by separately sampling from the leave-one-out posteriors via *N*_trials_ distinct MCMC runs. A more feasible approach comes from noting that all posteriors differ from the full posterior by only one data point. Therefore, the leave-one-out posteriors can be approximated via *importance sampling*, reweighting the full posterior obtained via MCMC. However, a direct approach of importance sampling can be unstable, since the full posterior is typically narrower than the leave-one-out posteriors. Pareto-smoothed importance sampling (PSIS) is a recent technique to stabilize the importance weights [52], implemented in the psisloo package (https://github.com/avehtari/PSIS). Thus, Eq 13 is approximated as

$$\begin{array}{c} \text{LOO}\approx \sum_{i=1}^{N_{\text{trials}}}\log\frac{\sum_{s=1}^{S}w_{i}^{\left(s\right)}p(r_{i}|\theta^{\left(s\right)})}{\sum_{s=1}^{S}w_{i}^{\left(s\right)}}, \end{array}$$ (14)

where ***θ***^(*s*)^ is the *s*-th parameter sample from the posterior, and $w_{i}^{\left(s\right)}$ are the Pareto-smoothed importance weights associated to the *i*-th trial and *s*-th sample (out of *S*); see [53] for details. PSIS also returns for each trial the exponent *k*_*i*_ of the fitted Pareto distribution; if *k*_*i*_ is greater than 1 the moments of the importance ratios distribution do not exist and the variance of the PSIS estimate is finite but may be large; this provides a natural diagnostic for the method [53] (see S1 Appendix). LOO is our comparison metric of choice (see Discussion). LOO scores for all models and subjects are reported in S1 Appendix.

Finally, we approximated the *marginal likelihood* of the model,

$$\begin{array}{c} p(\text{data}|\text{model})=\int p(\text{data}|\theta ,\text{model})p(\theta |\text{model})d\theta . \end{array}$$ (15)

The marginal likelihood is a common metric of model evidence that naturally incorporates a penalty for model complexity due to Bayesian Occam razor [71]. However, the integral in Eq 15 is notoriously hard to evaluate. Here we computed an approximation of the log marginal likelihood (LML) based on MCMC samples from the posterior, by using a *weighted* harmonic mean estimator [74]. The formula for the approximation is

$$\begin{array}{c} \text{LML}=-\log(\frac{1}{S}\sum_{s=1}^{S}\frac{\varphi (\theta^{\left(s\right)})}{p(\theta^{\left(s\right)})L(\theta^{\left(s\right)})}) \end{array}$$ (16)

where the sum is over *S* samples from the posterior, ***θ***^(*s*)^ is the *s*-th sample, *p*(***θ***) the prior, *L*(***θ***) the likelihood, and *φ*(***θ***) is an arbitrary weight probability density. The behavior of the approximation depends crucially on the choice of *φ*; it is important that *φ* has thinner tails than the posterior, lest the variance of the estimator grows unboundedly. We followed the suggestion of [74] and adopted a finite support distribution over a high posterior density region. We fitted a variational Gaussian mixture model to the posterior samples [98] (https://github.com/lacerbi/vbgmm), and then we replaced each Gaussian component with a uniform distribution over an ellipsoid region proportional to the covariance matrix of the component. The proportionality constant, common to all components, was picked by minimizing the empirical variance of the sum in Eq 16 [75].

#### Hierarchical Bayesian model selection

We performed Bayesian model selection at the group level via a hierarchical approach that treats subjects and models as random variables [54]. Group Bayesian Model Selection infers the posterior over model frequencies in the population, expressed as Dirichlet distributions parametrized by the concentration parameter vector ***α***. As a summary statistic we consider the protected exceedance probability $\tilde{\varphi }$, that is the probabilty that a given model or model factor is the most likely model or model factor, above and beyond chance [55]. For the *i*-th model or model factor,

$${\tilde{\varphi }}_{i}=\left(1-\text{BOR}\right)\varphi_{i}+\frac{1}{K}\text{BOR},$$

where *K* is the number of models (or model factors), *φ*_*i*_ is the unprotected exceedance probability for the *i*-th model or model factor [54], and BOR is the Bayesian omnibus risk—the posterior probability that the data may be explained by the null hypothesis according to which all models (or model factors) have equal probability [55]. For completeness, we report posterior model frequencies and BOR in the figures, but we do not focus on model frequencies per se since our sample size does not afford a more detailed population analysis.

To compute the posterior over model factors in the population we exploit the agglomerative propery of the Dirichlet distribution, and sum the concentration parameters of models that belong to the same factor component [54]. While the agglomerative property allows to easily compute the posterior frequencies and the *unprotected* exceedance probabilities for each model factor, calculation of the protected exceedance probabilities required us to compute the BOR for the model factor setup (the probability that the observed differences in factor frequencies may have arisen due to chance).

Additionally, the group Bayesian Model Selection method requires to specify a Dirichlet prior over model frequencies, represented by a concentration parameter vector *α*_0_ · ***w***, with *w*_*k*_ = 1 for any model *k* and *α*_0_ > 0. The common choice is *α*_0_ = 1 (flat prior over model frequencies), but given the nature of our factorial analysis we prefer a flat prior over model factors (*α*_0_ = average number factors / number of models), where the average number of factors is ≈ 2.33 for the bisensory tasks and ≈ 2.67 for the joint fits. This choice entails that the concentration parameter of the agglomerate Dirichlet distributions, obtained by grouping models that belong to the same factor component, is of order ∼1 (it cannot be exactly one since different factors have different number of components). When factor components within the same factor had unequal numbers of models, we modified the prior weight vector ***w*** such that every component had equal prior weight. We verified that our main results did not depend on the specific choice of Dirichlet prior (Fig 7, third row).

#### Parameter compatibility metric

Before performing the joint fits, we tested whether model parameters differed across the three tasks (unisensory and bisensory discrimination, unity judgment). On one end of the spectrum, the fully Bayesian approach would consist of comparing all combinations of models in which parameters are shared vs. distinct across tasks, and check which combination best explains the data. However, this approach is intractable in practice due to the combinatorial explosion of models, and undesirable in theory due to the risk model overfitting. On the simplest end of the spectrum, we could look at the credible intervals of the parameter posteriors for each subject and visually check whether they are mostly overlapping for different tasks.

As a middle ground, we computed separately for each parameter what we defined as the *compatibility probability* *C*_*p*_, that is the probability that for most subjects the parameter is exactly the same across tasks (*H*_0_), as opposed to being different (*H*_1_), above and beyond chance.

For a given subject, let *y*_1_, *y*_2_, and *y*_3_ be the datasets of the three tasks. For a given parameter *θ* (e.g., lapse rate), we computed the compatibility likelihoods

$$\begin{array}{c} \begin{array}{cc} p(y_{1},y_{2},y_{3}|H_{0})= & \int [\prod_{i=1}^{3}g_{i}\left(\theta |y_{i}\right)]f\left(\theta \right)d\theta ,\\ p(y_{1},y_{2},y_{3}|H_{1})= & \prod_{i=1}^{3}[\int g_{i}\left(\theta |y_{i}\right)f\left(\theta \right)d\theta ], \end{array} \end{array}$$ (17)

where *g*_*i*_(*θ*|*y*_*i*_) is the marginal posterior over *θ* for the dataset *y*_*i*_, and *f*(*θ*) is the prior over *θ*. Having computed the compatibility likelihoods for all subjects, we defined *C*_*p*_ as the protected exceedance probability of model *H*_0_ vs. model *H*_1_ for the entire group.

For each subject and task, the marginal posteriors *g*_*i*_(*θ*|*y*_*i*_) were obtained as a weighted average over models, with weight equal to each model’s posterior probability for that subject according to the group Bayesian Model Selection method via LOO, and considering only the subset of models that include the parameter of interest (see Fig 5).

For the prior *f*(*θ*) over a given parameter *θ*, for the purposes of this analysis only, we followed an empirical Bayes approach informed by the data and use a truncated Cauchy prior fitted to the average marginal posterior of *θ* across subjects, defined over the range of the MCMC samples for *θ*.

#### Absolute goodness of fit

Model comparison yields only a *relative* measure of goodness of fit, but does not convey any information of whether a model is a good description of the data in an absolute sense. A standard metric such as the coefficient of variation *R*^2^ is not appropriate for binary data. Instead, we extended the approach of [56] and defined *absolute goodness of fit* as

$$\begin{array}{c} g(\text{model})\equiv 1-\frac{{\hat{H}}_{G}\left(\text{data}\right)+\text{LOO}\left(\text{model}\right)}{{\hat{H}}_{G}\left(\text{data}\right)-N_{\text{trials}}\log2}, \end{array}$$ (18)

where ${\hat{H}}_{G}\left(\text{data}\right)$ is an estimate of the entropy of the data obtained via Grassberger’s estimator [99] and LOO(model) is the LOO score of the model of interest.

The numerator in Eq 18 represents the Kullback-Leibler (KL) divergence between the distribution of the data and the distribution predicted by the model (that is, how well the model captures the data), which is compared as a reference to the KL divergence between the data and a chance model (at the denominator). See S1 Appendix for a derivation of Eq 18, and code is available at https://github.com/lacerbi/gofit.

### The cookbook

The Bayesian cookbook for causal inference in multisensory perception, or simply ‘the cookbook’, consists of a recipe to build causal inference observer models for multisensory perception, and a number of algorithms and computational techniques to perform efficient and robust Bayesian comparison of such models. We applied and demonstrated these methods at different points in the main text; further details can be found here in the Methods and S1 Appendix. For reference, we summarize the main techniques of interest in Table 3.

**Table 3:** List of algorithms and computational procedures.

Description	Code	References
*Model fitting*		
Efficient computation of log likelihood	https://github.com/lacerbi/visvest-causinf	This work
Maximum-likelihood estimation (optimization)	https://github.com/lacerbi/bads	[78]
Posterior estimation (MCMC sampling)	https://github.com/lacerbi/eissample	In preparation
*Model evaluation and comparison*		
Leave-one-out cross validation (LOO)	https://github.com/avehtari/PSIS	[52, 53]
Estimate of the marginal likelihood	https://github.com/lacerbi/marglike	[74], in preparation
Parameter compatibility test	https://github.com/lacerbi/comprob	This work
Objective goodness of fit	https://github.com/lacerbi/gofit	[56], this work
Group Bayesian Model Selection	*spm_BMS* function in the SPM12 package http://www.fil.ion.ucl.ac.uk/spm/	[54, 55]

List of useful algorithms and computational procedures.

## Supporting information

S1 Fig Explicit causal inference; model fits of full data. Results of the explicit causal inference (unity judgment) task, for two models of interest. Proportion of ‘unity’ responses for a given (*s*_vis_, *s*_vest_) heading direction pair (indexed from 1 to 99), and for different levels of visual cue reliability. Points are data, lines are model fits (average fit across subjects). Error bars are omitted for clarity. **A**: Best Bayesian model (Bay-X-E). **B**: Best fixed-criterion model (Fix-C). Neither model appears clearly superior across all noise levels (see main text).
(TIF)

S1 Appendix Supplemental methods. Cookbook for causal inference observers. Observer model factors. Comparison between wrapped normal and von Mises noise. Computational details. Absolute goodness of fit. LOO scores for all subjects and models.
(PDF)
