# Supplementary figures and images for: Bayesian comparison of explicit and implicit causal inference strategies in multisensory heading perception
Source: PLoS Comput Biol. 2018 Jul 27;14(7):e1006110. doi: 10.1371/journal.pcbi.1006110 (PMC6063401; doi:10.1371/journal.pcbi.1006110)

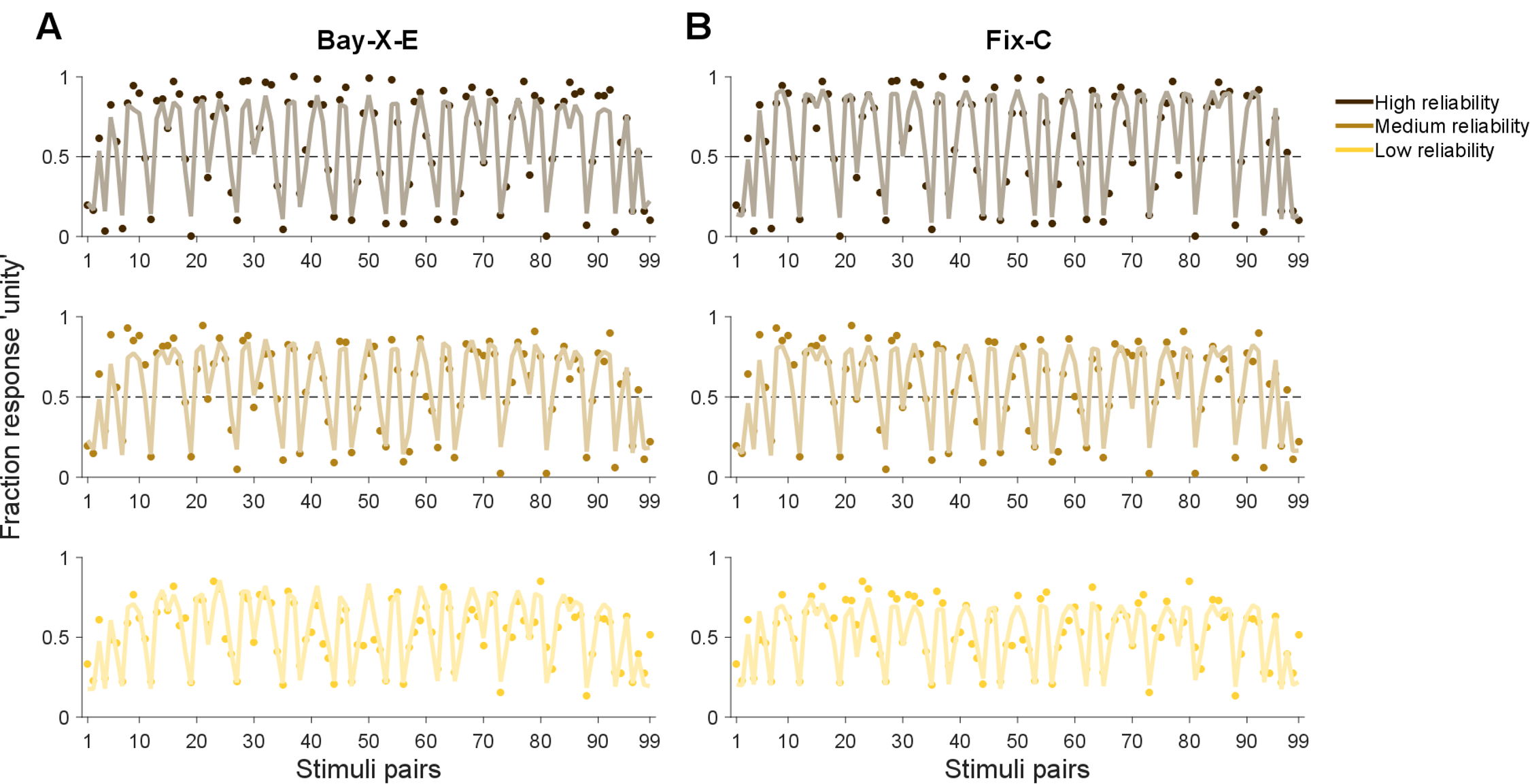

Supplement: S1 Fig — Results of the explicit causal inference (unity judgment) task, for two models of interest. Proportion of ‘unity’ responses for a given (svis, svest) heading direction pair (indexed from 1 to 99), and for different levels of visual cue reliability. Points are data, lines are model fits (average fit across subjects). Error bars are omitted for clarity. A: Best Bayesian model (Bay-X-E). B: Best fixed-criterion model (Fix-C). Neither model appears clearly superior across all noise levels (see main text). (TIF) [file pcbi.1006110.s001.tif]
