# Supplementary material for: Bayesian comparison of explicit and implicit causal inference strategies in multisensory heading perception
Source: PLoS Comput Biol. 2018 Jul 27;14(7):e1006110. doi: 10.1371/journal.pcbi.1006110 (PMC6063401; doi:10.1371/journal.pcbi.1006110)
Supplement: S1 Appendix — Cookbook for causal inference observers. Observer model factors. Comparison between wrapped normal and von Mises noise. Computational details. Absolute goodness of fit. LOO scores for all subjects and models. (PDF) [file pcbi.1006110.s002.pdf]

Bayesian Comparison of Explicit and Implicit Causal Inference Strategies in Multisensory Heading Perception

Appendix S1
Supplemental methods

Luigi Acerbi*, Kalpana Dokka*, Dora E. Angelaki, Wei Ji Ma

* These authors contributed equally to this work.
Contact: luigi.acerbi@{nyu.edu,gmail.com}

Contents

1 Cookbook for causal inference observers **2**
1.1 Pick a sensory noise model . . . 3
1.1.1 Measurement distribution and likelihoods . . . 3
1.2 Pick a prior over stimuli . . . 4
1.3 Pick a causal inference strategy . . . 4
1.3.1 Bayesian causal inference strategies . . . 4
1.3.2 Non-Bayesian causal inference strategies . . . 5
1.3.3 Non-causal inference strategies . . . 5
1.4 Pick other sources of suboptimality . . . 5

2 Observer model factors **6**
2.1 Sensory noise . . . 6
2.2 Prior . . . 6
2.3 Causal inference strategy . . . 7
2.4 Suboptimalities . . . 10
2.5 Model parameters . . . 10

3 Comparison between wrapped normal and von Mises noise **10**
3.1 Theoretical comparison . . . 10
3.2 Empirical comparison . . . 11

4 Computational details **11**
4.1 Integrals . . . 11
4.2 Optimization . . . 12
4.3 Markov Chain Monte Carlo (MCMC) sampling . . . 13
4.4 Pareto smoothed importance sampling diagnostics . . . 13
4.5 Visualization of model fits . . . 14
4.6 Model validation and recovery . . . 14

5 Absolute goodness of fit **16**
5.1 Computing the absolute goodness of fit 16
5.2 Entropy of the data 17
5.3 Cross-entropy 17

6 LOO scores for all models **18**
6.1 Unity judgment task 18
6.2 Bimodal inertial discrimination task 19
6.3 Joint fits 19

Supplemental References **19**

1 Cookbook for causal inference observers

We describe here a fairly general recipe for building an observer model for causal inference in multisensory perception. We consider the most common case of two sensory modalities (see [1] for work on three modalities). Stimuli take value on some one-dimensional physical continuum, such as location or heading direction.[1] The observer model is designed to apply to three types of tasks:

- *Unisensory estimation/discrimination*: The observer is presented with one stimulus from either modality, and is asked to report the value of the stimulus (or how the stimulus compares to a given reference).
- *Bisensory estimation/discrimination*: The observer is presented with two stimuli from different modalities, and is asked to report the value of either one, or of both (or how one of the stimuli, or both, compare to a given reference). Also referred to as *implicit* (causal) inference.
- *(Bisensory) unity judgement*: The observer is presented with two stimuli from different modalities, and is asked whether they were perceived as having the same value/source. Also referred to as *explicit* (causal) inference.

Depending on the experimental setup, the bisensory estimation/discrimination and unity judgment tasks might be performed in the same trial (a 'dual task' setup; see for example [2, 3]).

Our construction makes the following assumptions:

- When two stimuli are presented in the same trial, the observer follows a 'causal inference strategy' to decide whether the stimuli belong to a common cause ($C = 1$) or not ($C = 2$).
- Conditioned on a given causal scenario ($C = 1$ or $C = 2$), or in the unisensory task, the observer performs the estimation/discrimination task according to Bayesian inference.
- When responding, the observer might exhibit additional suboptimalities, such as lapsing and cue switching.

A specific observer model is built by picking four model components (also called model factors): (1) a sensory noise model; (2) a prior over stimuli; (3) a causal inference strategy; and (4) additional sources of suboptimality.

[1] With the risk of stating the obvious, we remark that stimulus value here is the quantity associated with the stimulus continuum and has nothing to do with 'value' in value-based decision making.

1.1 Pick a sensory noise model

For each modality 'mod', pick a sensory noise model for the observer. The common assumption is a Gaussian measurement noise distribution of the form

$$p(x_{\text{mod}}|s_{\text{mod}}) = \mathcal{N}\left(x_{\text{mod}}|s_{\text{mod}}, \sigma^2(s_{\text{mod}})\right), \tag{S1}$$

where x_{mod} is the noisy measurement, s_{mod} the stimulus value, $\mathcal{N}\left(x|\mu, \sigma^2\right)$ is a normal distribution with mean μ and variance σ^2, and the function $\sigma^2(s_{\text{mod}}) > 0$ encodes how the variance of measurement noise depends on the stimulus value, which is a feature of the sensory domain. Common shapes could be a constant noise, or alternatively noise that grows proportionally to $|s_{\text{mod}}|$ ($\sim$ Weber's law). The are no constraints on the shape of $\sigma^2(s_{\text{mod}})$ besides positivity and, arguably, continuity.

Eq. S1 is suitable for unbounded stimulus domains, or circular domains (such as orientation, or heading direction) with small angles, which effectively behave as unbounded domains. For an actually circular stimulus domain, we replace Eq. S1 with a wrapped normal distribution

$$p(x_{\text{mod}}|s_{\text{mod}}) = \sum_{k=-\infty}^{\infty} \mathcal{N}\left(x_{\text{mod}}|s_{\text{mod}} + 360°k, \sigma^2(s_{\text{mod}} + 360°k)\right), \qquad s_{\text{mod}} \in [-180°, 180°) \tag{S2}$$

which, for $\sigma(s_{\text{mod}}) < 360°$, is very well approximated by only three components $k = -1, 0, 1$. An alternative to Eq. S2 is to use a von Mises (i.e., circular normal) distribution; we show in Section 3 that the two choices are essentially equivalent.

1.1.1 Measurement distribution and likelihoods

We use Eqs. S1 (or S2) both for the *sensory measurement noise distribution*, that is the generative process of measurement x_{mod} for a given stimulus s_{mod} in the experiment, and for the observer's *sensory likelihood* used in the inference process of the posterior probability over s_{mod} for a given measurement x_{mod}. According to Bayes' rule, for the example of a unisensory stimulus, the latter takes the form

$$p(s_{\text{mod}}|x_{\text{mod}}) = \frac{p(x_{\text{mod}}|s_{\text{mod}})p_{\text{prior}}(s_{\text{mod}})}{\int p(x_{\text{mod}}|s'_{\text{mod}})p_{\text{prior}}(s'_{\text{mod}})ds'_{\text{mod}}} \tag{S3}$$

where $p_{\text{prior}}(s_{\text{mod}})$ is the prior over unisensory stimuli (see Section 1.2), and here $p(x_{\text{mod}}|s_{\text{mod}})$ is the likelihood.

Previous computational work has modified the equation of the measurement distribution by including terms, such as a scaling factor in front of x_{mod}, not reflected in the likelihood. This form of model mismatch has the effect of introducing *explicit* biases in subjects' percepts.[2] The rationale for this ad-hoc modification of the measurement distribution is that such biases are observed experimentally, see for example [4, 5] in the case of heading estimation. In our construction, instead, we follow the common practice in Bayesian psychophysics of assuming that biases in the observers' performance emerge *implicitly* and normatively from the interaction between statistics of the stimuli (i.e., priors) and precision of the sensory apparatuses (i.e., likelihoods) [6, 7]. Recent theoretical work has shown that this might agree with encoding of stimuli in neural populations [8, 9]. In particular, as demonstrated in these studies, priors will generally induce 'attractive' biases, whereas stimulus-dependent noise distributions (and, thus, likelihoods) can induce both 'attractive' and 'repulsive' biases. For this reason, we do not force biases by hand in the formulation of the sensory noise distribution, but this should not be mistaken for a lack of biases in the performance of our observer models.

[2] Note that if the same modifications were included in both measurement distribution and likelihood, they would 'cancel out' in the inference.

The fact that we use the same expressions (and parameters) for both the sensory measurement distribution and the likelihood is equivalent to saying that observers implicitly know their own noise model (that is, how noise changes as function of other parameters of the task, such as reliability and stimulus eccentricity). This modeling choice is motivated both by experimental work that shows trial-to-trial reweighing of multisensory cues [10–12] and by theoretical reasons, in that models in which noise (e.g., variance of measurement distributions) and beliefs about noise (e.g., 'variance' in the likelihoods) are decoupled may suffer from a lack of identifiability, unless the experiment is designed to avoid such issues [13].

1.2 Pick a prior over stimuli

The observer will have a prior over stimuli in the unisensory and bisensory conditions. A common choice for the prior is an independent, identical Gaussian prior across modalities for stimuli s_1 and s_2,

$$p_{\text{prior}}(s_1, s_2|C=2) = \mathcal{N}\left(s_1|\mu_{\text{prior}}, \sigma^2_{\text{prior}}\right)\mathcal{N}\left(s_2|\mu_{\text{prior}}, \sigma^2_{\text{prior}}\right) \tag{S4}$$

where μ_{prior} is the mean of the prior (which might represent a global bias, often assumed to be zero), and σ^2_{prior} represents the width of the prior (the wider the prior, the lesser its influence on behavior). The same prior is then applied to the common cause scenario and unisensory cases,

$$p_{\text{prior}}(s|C=1) = \mathcal{N}\left(s|\mu_{\text{prior}}, \sigma^2_{\text{prior}}\right). \tag{S5}$$

This simple prior induces a 'compression' or 'regression to the mean' bias as observed in many psychophysical experiments [14].

Another possibility is that the observer develops a prior (approximately) based on the empirical distribution of stimuli presented in the experiment, which may differ from Eqs. S4 and S5.

1.3 Pick a causal inference strategy

The causal inference strategy defines how the observer decides on the hypotheses $C = 1$ and $C = 2$ when presented with two stimuli. In general, the causal inference strategy may or may not be Bayesian, can be deterministic or stochastic, and might dictate to combine the two causal scenarios (e.g., by performing a weighted average of $C = 1$ and $C = 2$). This strategy also determines what the observer would report in an explicit, unity-judgment task.

1.3.1 Bayesian causal inference strategies

A Bayesian strategy will compute the posterior probabilities of the two causal scenarios, given the two noisy measurements x_1, x_2, as follows,

$$\begin{aligned} p(C|x_1, x_2) &\propto p(x_1, x_2|C)p(C) \\ &= p(C)\int p(x_1, x_2, s_1, s_2|C)ds_1 ds_2 \\ &= p(C)\int p(x_1|s_1)p(x_2|s_2)p_{\text{prior}}(s_1, s_2|C)ds_1 ds_2, \end{aligned} \tag{S6}$$

where $p(C)$ represents the prior belief of a common or separate cause, with $p(C = 1) = 1 - p(C = 2) \equiv p_{\text{c}}$. While p_{c} should typically stem from the statistics of the task, it is general practice to keep it as a free parameter of any Bayesian model, since subjects tend to exhibit a wide spectrum of beliefs about the probability of a common cause (see Fig 2 in [15]).

Different variants of Bayesian observers will use the posterior over causal scenarios differently to respond to estimation/discrimination task. Typical models are *Bayesian model averaging* (average the

estimates of $C = 1$ and $C = 2$, weighted by their posterior probability), *Bayesian model selection* (pick the estimate of either $C = 1$ or $C = 2$, based on which one has the larger posterior probability), or *Bayesian probability matching* (pick either scenario stochastically, with probability equal to their posterior probability).

For the unity judgment task, the standard Bayesian strategy is to respond with the scenario ($C = 1$ or $C = 2$) with highest posterior probability. Another possibility is posterior probability matching, that is pick either scenario stochastically, with probability equal to their posterior.

1.3.2 Non-Bayesian causal inference strategies

The main feature of a non-Bayesian strategy is that it does not compute a posterior distribution over causal scenarios, but uses instead (usually simpler) heuristics as a decision rule to whether $C = 1$ or $C = 2$.

A typical heuristic of this kind stipulates that $C = 1$ whenever the two noisy measurements x_1, x_2 are closer in value than some criterion κ, that is $|x_1 - x_2| < \kappa$. If κ is fixed for all experimental conditions, we call this a *fixed-criterion* causal inference strategy [16]. If κ is allowed to change for different experimental conditions, and in particular as a function of stimulus reliability, then the decision rule becomes 'probabilistic', that is uncertainty-dependent [17].

A fixed-criterion strategy that discards reliability information might seem to clash with the assumption that observers know the stimulus reliability when combining cues. However, there is neural evidence that sensory integration (that is forced fusion, with reliability-dependent weighing) and causal inference happen in different brain areas [18]. For this reason, it is not obvious that reliability information would be automatically available to higher areas, or that it would be used in the correct way. Fixed-criterion models represent a valid 'null' alternative for a class of models in which reliability information is unavailable (or corrupted) at the causal inference stage.

1.3.3 Non-causal inference strategies

Extreme cases of causal inference strategies are observers that do not quite perform causal inference at all.

In this case, an observer might use a *forced fusion* strategy that always combines cues ($C \equiv 1$), or, alternatively, a *forced segregation* strategy that always segregates them ($C \equiv 2$). Mathematically, these strategies can be considered as limiting cases of previously presented causal inference strategies. For example, forced fusion is equivalent to a Bayesian causal inference strategy with $p_c \to 1$, or a fixed-criterion strategy with $\kappa \to \infty$. Analogously, forced segregation is equivalent to a Bayesian strategy with $p_c \to 0$, or a fixed-criterion strategy with $\kappa \to 0$.

As a generalization of forced fusion/segregation, we can consider a *stochastic fusion* strategy that on each trial has probability η of deciding $C = 1$, and $C = 2$ otherwise, where η might depend on the experimental condition.

1.4 Pick other sources of suboptimality

Experimental subjects will often exhibit additional sources of variability, which might be included explicitly in the model. Here we consider lapses and cue switching.

A common feature of many psychophysical models is a *lapse rate*, that is the probability λ that the observer gives a completely random response (typically, uniform over the range of possible responses) [19].

Another form of error for multisensory perception experiments is that the observer switches modality, that is in a bisensory estimation/discrimination task they respond about the wrong modality with *switching rates* $\rho_{1\to2}$ and $\rho_{2\to1}$, respectively for responding with the second modality when asked about the first, and vice versa. Note that the switching rate can be used to implement suboptimal strategies

such as *cue capture*, whereby all responses are absorbed by a single modality: pick the 'forced segregation' causal inference strategy, then set, say, $\rho_{2\to 1} = 1$ and $\rho_{1\to 2} = 0$, if responses are supposed to be captured by the first modality. Similarly, by picking 'forced segregation' with nonzero $\rho_{1\to 2}$ and $\rho_{2\to 1}$, one can implement a *switching strategy* observer [20].

2 Observer model factors

In this section we describe details of the factors used to build the observer models in the paper.

2.1 Sensory noise

For a given modality mod $\in \{\text{vis}, \text{vest}\}$, the measurement noise distribution follows Eq. S1. Note that for a visual stimulus the measurement distribution and the variance in Eq. S1 also depend on the visual coherence level c_{vis} in the trial, such that $\sigma^2(s_{\text{vis}}) \equiv \sigma^2(s_{\text{vis}}, c_{\text{vis}})$, but in the following we omit this dependence to simplify the notation.

For the variance we consider two possible models,

$$\sigma^2(s_{\text{mod}}) = \begin{cases} \sigma^2_{0\,\text{mod}} & \text{(constant)} \\ \sigma^2_{0\,\text{mod}} \left\{ 1 + 2 w^2_{\text{mod}} \left(\frac{90^\circ}{\pi} \right)^2 \left[1 - \cos\left(\frac{s_{\text{mod}}}{90^\circ} \pi \right) \right] \right\} & \text{(eccentricity-dependent)} \end{cases} \tag{S7}$$

where $\sigma^2_{0\,\text{modality}}$ is the base variance and w_{mod} is related to the Weber fraction near 0°. In fact, for small values of s_{mod}, Eq. S7 reduces to $\sigma^2(s_{\text{mod}}) \approx \sigma^2_{0\,\text{mod}} \left(1 + w^2_{\text{mod}} s^2_{\text{mod}} \right)$, which is a generalized Weber's law.[3]

The broad shape of the chosen periodic formula for the eccentriticy-dependent noise model, which peaks at $\pm 90^\circ$, derives from empirical results in a visuo-vestibular task with the same apparatus with human and monkey subjects (see Fig 2 in [4]; see also [22]). We note that our noise shape differs from that adopted in other works (with different setups), which used a sinusoidal with twice the frequency that peaks at $\pm 45^\circ, \pm 135^\circ$ [23, 24]. Since in our setup the heading directions were restricted to the $\pm 45^\circ$ range (with most directions in the $\pm 25^\circ$ range), the exact shape of periodicity is largely irrelevant, but understanding differences in noise models may be important for experiments with wider heading direction ranges.

For the paper, we implemented the measurement distribution (and, thus, the stimulus likelihood in the inference process) as a mixture of three wrapped Gaussians (Eq. S2). However, we found that, due to the limited range of directions in our experiment, a single Gaussian was sufficient. Note that our choice of using Gaussians rather than von Mises (circular normal) distributions yields no loss of generality in practice, as we demonstrate in Section 3.

All constant noise models have four parameters ($\sigma_{0\text{vest}}$, and a separate $\sigma_{0\text{vis}}$ for each visual coherence level, low, medium and high). Eccentricity-dependent models have two additional parameters, w_{vest} and w_{vis} (the latter is common to all visual stimuli, to prevent overfitting).

2.2 Prior

For unisensory trials, we assume that observers have a unimodal symmetric prior over heading directions, peaked at 0° (the exact shape is irrelevant). Due to the form of the decision rule for the left/right discrimination task, such prior has no influence over the observer's response, which only depends on whether the noisy measurement falls to the left or to the right of straight ahead.

[3] Here by Weber's law we simply denote the fact that noise scales proportionally to stimulus magnitude, that is $\sigma(s) \propto |s|$. Technically, Weber's law is defined only for quantity-related continua, whereas heading is a quality-related continuum [21].

For bisensory trials (both unity judgment and inertial discrimination tasks), we consider two alternative models for priors. The *empirical* prior consists of an approximation of the actual prior used in the experiment, that is

$$\begin{aligned} p(s_{\text{vis}}, s_{\text{vest}}|C=1) &\propto \sum_{(s,s)\in\mathcal{S}} \mathcal{N}\left(s|0,\sigma^2_{\text{prior}}\right)\delta(s_{\text{vis}}-s)\delta(s_{\text{vest}}-s) \\ p(s_{\text{vis}}, s_{\text{vest}}|C=2) &\propto \sum_{\substack{(s_i,s_j)\in\mathcal{S} \\ s_i\neq s_j}} \mathcal{N}\left(\frac{s_{\text{vis}}+s_{\text{vest}}}{2}|0,\sigma^2_{\text{prior}}\right)\mathcal{N}\left(s_{\text{vest}}-s_{\text{vis}}|0,\Delta^2_{\text{prior}}\right)\delta(s_{\text{vis}}-s_i)\delta(s_{\text{vest}}-s_j) \end{aligned} \tag{S8}$$

where $\mathcal{S}$ is the discrete set of pairs of visual and vestibular headings in the experiment. The two equations consider respectively only diagonal elements (equal heading directions, $C = 1$) or off-diagonal elements (different directions, $C = 2$) of Fig 1B in the main text. The approximation here is given by the two Gaussian distributions (defined on the discrete set), which impose additional shrinkage for the mean of the stimuli (governed by σ^2_{prior}) and for the disparity (governed by Δ^2_{prior}). For $\sigma^2_{\text{prior}}, \Delta^2_{\text{prior}} \to \infty$, Eq. S8 converges to the distributions of directions used in the experiment for $C = 1$ and $C = 2$.

Alternatively, we consider an *independent* prior, that is

$$\begin{aligned} p(s_{\text{vis}}, s_{\text{vest}}|C=1) &= \int \mathcal{N}\left(s|0,\sigma^2_{\text{prior}}\right)\delta(s_{\text{vis}}-s)\delta(s_{\text{vest}}-s)ds \\ p(s_{\text{vis}}, s_{\text{vest}}|C=2) &= \mathcal{N}\left(s_{\text{vis}}|0,\sigma^2_{\text{prior}}\right)\mathcal{N}\left(s_{\text{vest}}|0,\sigma^2_{\text{prior}}\right) \end{aligned} \tag{S9}$$

which assumes observers build a single prior over heading directions which is applied independently to both modalities [25]. The first integral is a formal way to impose $s \equiv s_{\text{vis}} = s_{\text{vest}}$.

We note that a continuous approximation of Eq. S8 may seem more realistic than the adopted discrete distribution of directions. However, an observer model with a correlated, continuous prior is computationally intractable since evaluation of the log likelihood involves a non-analytical four-dimensional integral, which increases the computational burden by an order of magnitude. As a sanity check, we implemented observers that use a continuous approximation of Eq. S8 and verified on a subset of observers and models that results of model fits and model predictions were indeed nearly identical to the discrete case.

Independent prior models have one parameter σ_{prior} for the width of the prior over headings. Empirical prior models have an additional parameter Δ_{prior} for the width of the prior over disparities.

2.3 Causal inference strategy

The basic causal inference strategies: *Bayesian*, *fixed-criterion* and *fusion* are described in the main text. We report here some additional definitions and derivations.

All integrals in this section are in the $[-90°, 90°]$ range, unless noted otherwise. The rationale of such integration range for our experiment is that subjects were informed that the movement was forward (either left or right of straight-forward). Moreover, due to the relatively narrow range of stimuli used in our experiment, we found with preliminary analyses that beliefs more than 90° away from straight-ahead had negligible influence on left/right decisions. In the more general case of stimuli distributed along the full circle, the integration range should go to ±180°. For a non-circular dimension, appropriate empirical bounds should be chosen (e.g., the width of the projection screen for a localization task).

Posterior probability of causal structure

For a Bayesian observer, the posterior probability of common cause is

$$\Pr(C=1|x_{\text{vest}}, x_{\text{vis}}, c_{\text{vis}}) \propto p(x_{\text{vis}}, x_{\text{vest}}, c_{\text{vis}}|C=1)\Pr(C=1)$$

where $\Pr(C=1) \equiv p_c$, the prior probability of a common cause, is a free parameter of the model. Then

$$\begin{aligned} p(x_{\text{vis}}, x_{\text{vest}}, c_{\text{vis}} | C = 1) = \\ = \Pr(c_{\text{vis}}) \int\int p(x_{\text{vis}}|s_{\text{vis}}, c_{\text{vis}}) p(x_{\text{vest}}|s_{\text{vest}}) p(s_{\text{vis}}, s_{\text{vest}}|C=1) ds_{\text{vis}} ds_{\text{vest}}, \end{aligned} \tag{S10}$$

where the likelihoods are defined by Eq. S1, the prior is defined by Eqs. S8 and S9, and $\Pr(c_{\text{vis}}) = \frac{1}{3}$. For the independent prior case we can further simplify

$$p(x_{\text{vis}}, x_{\text{vest}}, c_{\text{vis}} | C = 1) \propto \int p(x_{\text{vis}}|s_{\text{vis}} = s_{\text{vest}}, c_{\text{vis}}) p(x_{\text{vest}}|s_{\text{vest}}) \mathcal{N}\left(s_{\text{vest}}|0, \sigma^2_{\text{prior}}\right) ds_{\text{vest}},$$

whereas the solution for the empirical prior is similar, but with a sum over the discrete stimuli such that $s_{\text{vis}} = s_{\text{vest}}$.

Conversely, the posterior probability of separate causes is

$$\Pr(C = 2|x_{\text{vis}}, x_{\text{vest}}, c_{\text{vis}}) \propto p(x_{\text{vis}}, x_{\text{vest}}, c_{\text{vis}}|C = 2)\left(1 - p_c\right),$$

where

$$p(x_{\text{vis}}, x_{\text{vest}}, c_{\text{vis}} | C = 2) = \Pr(c_{\text{vis}}) \int\int p(x_{\text{vis}}|s_{\text{vis}}, c_{\text{vis}}) p(x_{\text{vest}}|s_{\text{vest}}) p(s_{\text{vis}}, s_{\text{vest}}|C=2) ds_{\text{vis}} ds_{\text{vest}}, \tag{S11}$$

which for the independent prior becomes

$$p(x_{\text{vis}}, x_{\text{vest}}, c_{\text{vis}} | C = 2) \propto \left(\int p(x_{\text{vis}}|s_{\text{vis}}, c_{\text{vis}}) p_{\text{prior}}(s_{\text{vis}}) ds_{\text{vis}}\right) \cdot \left(\int p(x_{\text{vest}}|s_{\text{vest}}) p_{\text{prior}}(s_{\text{vest}}) ds_{\text{vest}}\right),$$

that is the product of two one-dimensional integrals. For the empirical prior Eq. S11 does not simplify, but becomes a discrete sum over $\mathcal{S}$ (see Eq. S8).

Posterior probability of left/right discrimination ($C = 1$)

In bisensory inertial discrimination trials the observer may implicitly contemplate two scenarios: that there is only one common cause ($C = 1$), or that there are two distinct causes ($C = 2$). We consider inference in the two separate scenarios, and then see how the observer can combine them.

For $C = 1$, the observer's posterior probability density over over the inertial heading direction is

$$\begin{aligned} p\left(s_{\text{vest}}|x_{\text{vis}}, x_{\text{vest}}, c_{\text{vis}}, C = 1\right) = \\ = \int \frac{p(s_{\text{vis}}, s_{\text{vest}}, x_{\text{vis}}, x_{\text{vest}}, c_{\text{vis}}, C = 1)}{p(x_{\text{vis}}, x_{\text{vest}}, c_{\text{vis}}, C = 1)} ds_{\text{vis}} \\ = \int \frac{p(s_{\text{vis}}, s_{\text{vest}}, x_{\text{vis}}, x_{\text{vest}}, c_{\text{vis}}|C = 1)\Pr(C = 1)}{p(x_{\text{vis}}, x_{\text{vest}}, c_{\text{vis}}|C = 1)\Pr(C = 1)} ds_{\text{vis}} \\ \propto \int p(x_{\text{vest}}|s_{\text{vest}}) p(x_{\text{vis}}|s_{\text{vis}}, c_{\text{vis}}) p(s_{\text{vis}}, s_{\text{vest}}|C = 1) ds_{\text{vis}} \end{aligned} \tag{S12}$$

which for the independent prior becomes

$$p\left(s_{\text{vest}}|x_{\text{vis}}, x_{\text{vest}}, c_{\text{vis}}, C = 1\right) \propto p(x_{\text{vest}}|s_{\text{vest}}) p(x_{\text{vis}}|s_{\text{vis}} = s_{\text{vest}}, c_{\text{vis}}) \mathcal{N}\left(s_{\text{vest}}|0, \sigma^2_{\text{prior}}\right)$$

and the solution is similar for the empirical prior, constraining s_{vest} to take only the discrete values used in the experiment for $C = 1$.

Posterior probability of left/right discrimination ($C = 2$)

For $C = 2$, the observer's posterior over inertial heading is

$$\begin{aligned} & p\left(s_{\text{vest}} | x_{\text{vis}}, x_{\text{vest}}, c_{\text{vis}}, C = 2\right) = \\ & \quad = \int \frac{p(s_{\text{vis}}, s_{\text{vest}}, x_{\text{vis}}, x_{\text{vest}}, c_{\text{vis}}, C = 2)}{p(x_{\text{vis}}, x_{\text{vest}}, c_{\text{vis}}, C = 2)} ds_{\text{vis}} \\ & \quad \propto \int p(x_{\text{vest}}|s_{\text{vest}}) p(x_{\text{vis}}|s_{\text{vis}}) p(s_{\text{vis}}, s_{\text{vest}}|C = 2) ds_{\text{vis}} \end{aligned} \tag{S13}$$

which for the independent prior can be further simplified as

$$p\left(s_{\text{vest}} | x_{\text{vis}}, x_{\text{vest}}, c_{\text{vis}}, C = 2\right) \propto p(x_{\text{vest}}|s_{\text{vest}}) \mathcal{N}\left(s_{\text{vest}}|0, \sigma^2_{\text{prior}}\right),$$

whereas for the empirical prior the integral in Eq. S13 becomes a sum over discrete pairs of heading directions used in the experiment.

Posterior probability of left/right discrimination (C unknown)

If the causal structure is unknown, a Bayesian observer that follows a 'model averaging' strategy marginalizes over possible causal structures (here, $C = 1$ and $C = 2$) [25]. The observer's posterior probability density over the inertial heading direction is

$$\begin{aligned} & p\left(s_{\text{vest}} | x_{\text{vis}}, x_{\text{vest}}, c_{\text{vis}}\right) = \\ & \quad = \sum_{C=1,2} \int \frac{p(s_{\text{vis}}, s_{\text{vest}}, x_{\text{vis}}, x_{\text{vest}}, c_{\text{vis}}, C)}{p(x_{\text{vis}}, x_{\text{vest}}, c_{\text{vis}})} ds_{\text{vis}} \\ & \quad = \frac{1}{p(x_{\text{vis}}, x_{\text{vest}}, c_{\text{vis}})} \left[\int p(s_{\text{vis}}, s_{\text{vest}}, x_{\text{vis}}, x_{\text{vest}}, c_{\text{vis}}, C = 1) ds_{\text{vis}} + \right. \\ & \quad \left. \int p(s_{\text{vis}}, s_{\text{vest}}, x_{\text{vis}}, x_{\text{vest}}, c_{\text{vis}}, C = 2) ds_{\text{vis}} \right] \\ & \quad = \frac{p(x_{\text{vis}}, x_{\text{vest}}, c_{\text{vis}}, C = 1)}{p(x_{\text{vis}}, x_{\text{vest}}, c_{\text{vis}})} p(s_{\text{vest}}|x_{\text{vis}}, x_{\text{vest}}, c_{\text{vis}}, C = 1) + \\ & \quad \frac{p(x_{\text{vis}}, x_{\text{vest}}, c_{\text{vis}}, C = 2)}{p(x_{\text{vis}}, x_{\text{vest}}, c_{\text{vis}})} p(s_{\text{vest}}|x_{\text{vis}}, x_{\text{vest}}, c_{\text{vis}}, C = 2) \\ & \quad = \Pr(C = 1|x_{\text{vis}}, x_{\text{vest}}, c_{\text{vis}}) \cdot p(s_{\text{vest}}|x_{\text{vis}}, x_{\text{vest}}, c_{\text{vis}}, C = 1) + \\ & \quad \Pr(C = 2|x_{\text{vis}}, x_{\text{vest}}, c_{\text{vis}}) \cdot p(s_{\text{vest}}|x_{\text{vis}}, x_{\text{vest}}, c_{\text{vis}}, C = 2) \end{aligned} \tag{S14}$$

where $p(s_{\text{vest}}|x_{\text{vis}}, x_{\text{vest}}, c_{\text{vis}}, C)$ has been defined in the previous subsections and $\Pr(C|x_{\text{vis}}, x_{\text{vest}}, c_{\text{vis}})$ is the posterior over causal structures.

We generalize Eq. S14 as

$$\begin{aligned} p\left(s_{\text{vest}} | x_{\text{vis}}, x_{\text{vest}}, c_{\text{vis}}\right) = & v_1(x_{\text{vis}}, x_{\text{vest}}, c_{\text{vis}}) \cdot p(s_{\text{vest}}|x_{\text{vis}}, x_{\text{vest}}, c_{\text{vis}}, C = 1) + \\ & v_2(x_{\text{vis}}, x_{\text{vest}}, c_{\text{vis}}) \cdot p(s_{\text{vest}}|x_{\text{vis}}, x_{\text{vest}}, c_{\text{vis}}, C = 2) \end{aligned}$$

where $v_k(x_{\text{vis}}, x_{\text{vest}}, c_{\text{vis}})$, for $k = 1, 2$, are the posterior *causal weights* assigned by the observer to the two causal structures, with $v_2(x_{\text{vis}}, x_{\text{vest}}, c_{\text{vis}}) = 1 - v_1(x_{\text{vis}}, x_{\text{vest}})$ and $0 \leq v_1(x_{\text{vis}}, x_{\text{vest}}, c_{\text{vis}}) \leq 1$. For a Bayesian observer, the causal weights are equal to the posterior probabilities (Eq. S14); in the main text we describe other models.

2.4 Suboptimalities

For all our observer models, we considered a lapse rate λ. Due to the format of our bisensory discrimination data (i.e., only inertial left/right responses), which limits the identifiability of switching models, we did not consider a switching rate, leaving that to future work.

2.5 Model parameters

All models except stochastic fusion have five parameters $\boldsymbol{\theta}_{\text{default}}$ by default: three visual base noise parameters $\sigma_{0\text{vis}}(c_{\text{high}})$, $\sigma_{0\text{vis}}(c_{\text{med}})$, and $\sigma_{0\text{vis}}(c_{\text{low}})$; a vestibular base noise parameter $\sigma_{0\text{vest}}$; and a lapse rate λ.

**Observer model**	**Parameters**	**#**
Bayesian (*unisensory only*)	$\boldsymbol{\theta}_{\text{default}}$	5
Bayesian causal inference	$\boldsymbol{\theta}_{\text{default}}, \sigma_{\text{prior}}, p_{\text{c}}$	7
Fixed-criterion causal inference	$\boldsymbol{\theta}_{\text{default}}, \kappa_{\text{c}}$	6
Fusion causal inference	$\boldsymbol{\theta}_{\text{default}}$	5
Stochastic fusion (*unity judgment only*)	$\eta_{\text{high}}, \eta_{\text{med}}, \eta_{\text{low}}$	3
Add-ons		
with eccentricity-dependent noise	$+\{w_{\text{vis}}, w_{\text{vest}}\}$	+2
with empirical priors (*Bayesian*)	$+\{\Delta_{\text{prior}}\}$	+1
with empirical priors (*non-Bayesian*)	$+\{\sigma_{\text{prior}}, \Delta_{\text{prior}}\}$	+2

3 Comparison between wrapped normal and von Mises noise

In the presentation of our general causal inference observer model, and in the manuscript, we assumed that measurement noise distributions took the shape of (wrapped) normals (see Eqs. S1 and S2). Moreover, for wrapped normals, we advocated that three mixture components ($k = 0, \pm 1$) are sufficient. Our modeling proposal differs from the typical choice of using von Mises (circular normal) distributions for circular variables (see for example [23, 24]). Here we test whether our choice is sensible and generally applicable, by asking whether there is a practical difference between using von Mises and wrapped normals, for experiments with stimuli over the entire circular domain.

First, we note that, qualitatively, the von Mises and wrapped normals have very similar properties. They are both bell-shaped distributions over the circle, and they are both related to the normal distribution. von Mises distributions are the maximum-entropy distributions over the circle, so theoretically more appealing, but on the other hand wrapped normals, especially as a mixture of three Gaussians (one at the mean, the other two at $\pm 360°$ from the mean), have computational advantages. It remains to be established whether these distributions differ quantitatively in an empirically meaningful way. In the following analyses, we always consider wrapped normals approximated with three mixture components.

3.1 Theoretical comparison

To answer this question theoretically, we assess the difference between the two noise distributions by computing the Kullback-Leibler (KL) divergence between a von Mises distribution with a given concentration parameter κ and the best approximating wrapped normal (this construction assumes that the true underlying distribution is a von Mises, but the results are similar after inverting the role). The KL-divergence represents the expected difference in log likelihood between the two noise models per trial (assuming the data were generated from a von Mises). Thus, the *inverse* of the KL-divergence can be taken as a ballpark of the minimum number of samples required to empirically see a difference between the two models (that is, one point of log likelihood of difference summed over trials). We call this quantity the *identifiability threshold*.

As expected, for large values of κ (when the von Mises converges to a normal distribution) and for small values of κ (when the von Mises converges to a uniform distribution over the circle), the identifiability threshold between wrapped normal and von Mises is way over 10^3, and even 10^4, meaning that several thousand trials would be needed to distinguish the two models (assuming no other confounding elements). However, there is a range of values of κ, around $\approx$ 50-60 (that is, a circular SD of $\approx 7°$), in which the identifiability threshold drops to $\approx$ 60-100. This analysis tells that, at least in some cases, the models *could* be distinguished within a large but feasible amount of trials. Whether the two noise models can be distinguished in practice is an empirical question, since in real data differences in the noise models will be obfuscated by other details. Moreover, it is possible that neither model is the true one (but they could be both equally good at approximating the true model). Finally, subjects' typical parameters might reside in ranges in which the two distributions are not empirically distinguishable.

3.2 Empirical comparison

To answer this question empirically, we took the data from a recent paper on causal inference in multisensory heading estimation [24]. For all subjects (17 datasets between Experiment 1 and 2, 400-600 trials per dataset), we fit the unisensory data (four conditions: one visual and three inertial) using the basic modeling framework described in the section "Analyses of Unisensory Data" of [24]. One minor difference with their analysis is that, as a principled way of dealing with outliers, we added for each subject a lapse rate parameter, shared across conditions (instead of discarding data points more than three standard deviations away from the mean). The lapse rate represents the probability of a completely random response (e.g., due to a missed stimulus, or a mistake in the response).

Crucially, we considered two models, one in which the noise model is a von Mises (as per [24]), and another one in which the noise model is a wrapped Gaussian (implemented as a mixture of Gaussians with three components). We fitted each dataset to both models via maximum-likelihood estimation. For the optimization, we used MATLAB's `fmincon` function with 100 random restarts, plus one starting point represented by the maximum-likelihood solution reported in [24, S2 Table]. Since both models have the same number of parameters (and, moreover, all parameters have the same meaning), we can directly compare differences in log likelihood without the need to account for model complexity. Across subjects, we found a difference of log likelihood of 0.13 ± 0.18 (mean $\pm$ S.E.M.), which is negligible evidence in favor of the von Mises distribution. In fact, most of the evidence comes from a single subject; otherwise, eight subjects slightly favor the wrapped normal, and other eight slightly favor the von Mises. These results show that the two models are practically indistinguishable in real continuous estimation data. Note that this would be even more so with our data, since we have only discrete (binary) responses.

In conclusion, these analyses support our choice of using (wrapped) normals as an equivalent alternative to von Mises distributions, and suggest that wrapped normals, approximated via three mixture components, could be used more generally as a valid computational alternative to von Mises distributions.

4 Computational details

We describe in this section a number of computational and algorithmic details.

4.1 Integrals

Due to lack of analytical solutions, we computed all one-dimensional and two-dimensional integrals numerically, via either Simpson's or trapezoidal rule with a equi-spaced grid on the integration domain [26]. We had two types of integrals: integrals over x_{vis}, x_{vest} for marginalization over the noisy stimuli, and integrals over s_{vis} and/or s_{vest} for computation of the observer's decision rule (Eqs. S10, S11, S12 and S13).

For marginalization over noisy measurement x_{vis} and x_{vest}, we used a regular 401×401 grid for which we adjusted the range of integration in each modality to up to 5 SD from the mean of the noisy measurement distribution (or $\pm 180°$, whichever was smaller). For large noise, we used wrapped normal distributions, which turned out to have little effect due to our setup.

For computation of the decision rule, we assumed that observers believed, due to the experimental setup and task instructions, that the movement direction would be forward, so limited to the $\pm 90°$ range. We adjusted the integration grid spacing Δs (hence the number of grid points) adaptively for each parameter vector $\boldsymbol{\theta}$, defining

$$\begin{aligned}\sigma_{\min}(\boldsymbol{\theta}, c_{\text{vis}}) &= \min\left\{\sigma_{0\text{vis}}(c_{\text{vis}}), \sigma_{0\text{vest}}, \sigma_{\text{prior}}\right\} \\ \Delta s &\equiv \frac{\sigma_{\min}(\boldsymbol{\theta}, c_{\text{vis}})}{4} \qquad \text{with} \quad \frac{1}{8} \leq \Delta s \leq 1\end{aligned}$$

and we rounded Δs to the lowest exact fraction of the form $\frac{1}{m}$, with $m \in \mathbb{N}$ and $1 \leq m \leq 8$. The above heuristic afforded fast and accurate evaluation of the integrals, since the grid spacing was calibrated to be smaller than the length scale of the involved distributions (measurement noise and prior).

Finally, we note that we tried other standard numerical integration methods which were ineffective. Gauss-Hermite quadrature [26] led to large numerical errors because the integrand is discontinuous and bounded, a very bad fit for a polynomial. Global adaptive quadrature methods (such as `quad` in MATLAB, and other custom-made implementations) were simply too slow, even when reducing the requested precision. We coded all two-dimensional numerical integrals in C (via *mex* files in MATLAB) for maximal performance.

4.2 Optimization

For optimization of the log likelihood (maximum-likelihood estimation), we used Bayesian Adaptive Direct Search (BADS [27]; `https://github.com/lacerbi/bads`). BADS follows a mesh adaptive direct search (MADS) procedure that alternates POLL steps and SEARCH steps. In the POLL step, points are evaluated on a (random) mesh by taking one step in one coordinate direction at a time, until an improvement is found or all directions have been tried. The step size is doubled in case of success, halved otherwise. In the SEARCH step, a Gaussian process is fit to a (local) subset of the points evaluated so far. Points to evaluate during the search are iteratively chosen by maximizing the predicted improvement (with respect to the current optimum) over a set of candidate points. Adherence to the MADS framework guarrantees convergence to a (local) stationary point of a noiseless function under general conditions [28]. The basic scheme is enhanced with heuristics to accelerate the POLL step, to update the Gaussian process hyperparameters, to generate a good set of candidate points in the search step, and to deal robustly with noisy functions. See [27] for details.

For each optimization run, we initialized our algorithm by randomly choosing a point inside a hypercube of plausible parameter values in parameter space. We refined the output of each BADS run with a run of `patternsearch` (MATLAB). To avoid local optima, for each optimization problem we performed 150 independent restarts of the whole procedure and picked the highest log likelihood value.

As a heuristic diagnostic of global convergence, we computed by bootstrap the value of the global optimum we would have found had we only used n_{r} restarts, with $1 \leq n_{\text{r}} \leq 150$. We define the 'estimated regret' as the difference between the actual best value of the log likelihood found and the bootstrapped optimum. For each optimization problem, we computed the minimum value n_{r}^* for which the probability of having an estimated regret less than 1 was 99% ($n_{\text{r}}^* \equiv \infty$ if such n_{r} does not exist). The rationale is that if the optimization landscape presents a large number of local optima, and new substantially improved optima keep being found with increasing n_{r}, the bootstrapped estimated regret would keep changing with n_{r}, and n_{r}^* would be 150 or ∞. For almost all optimization problems, we found $n_{\text{r}}^* \ll 150$. This suggests that the number of restarts was large enough; although no optimization procedure in a non-convex setting can guarantee convergence to a global optimum in a finite time without further assumptions.

4.3 Markov Chain Monte Carlo (MCMC) sampling

As a complementary approach to maximum-likelihood model fitting, for each dataset and model we calculated the posterior distribution of the parameters via MCMC (see main text).

We used a custom-written sampling algorithm that combines slice sampling [29] with adaptive direction sampling [30].[4] Slice sampling is a flexible MCMC method that, in contrast with the common Metropolis-Hastings transition operator, requires very little tuning in the choice of length scale. Adaptive direction sampling is an ensemble MCMC method that shares information between several dependent chains (also called 'walkers' [31]) in order to speed up mixing and exploration of the state space. For each ensemble we used $2(p+1)$ walkers, where p is the number of parameters of the model. Walkers were initialized to a neighborhood of the best local optima found by the optimization algorithm. Each ensamble was run for 10^4 to $2.5 \cdot 10^4$ burn-in steps that were discarded, after which we collected $5 \cdot 10^3$ to 10^4 samples per ensemble.

At each step, our method iteratively selects one walker in the ensemble and first attempts an independent Metropolis update. The proposal distribution for the independent Metropolis is a variational mixture of Gaussians [32] fitted to a fraction of the samples obtained during burn-in via the `vbgmm` toolbox for MATLAB.[5] Note that the proposal distribution is fixed at the end of burn-in and does not change thereafter, ensuring that the Markov property is not affected (although non-Markovian adaptive MCMC methods could be applied; see [33]). After the Metropolis step, the method randomly applies with probability 1/3 one of three Markov transition operators to the active walker: coordinate-wise slice sampling [29], parallel-direction slice sampling [34], and adaptive-direction slice sampling [29, 30]. We also fit a variational Gaussian mixture model to the last third of the samples at the end of the burnin period, and we used the variational mixture as a proposal distribution for an independent Metropolis step which was attempted at every step.

For each dataset and model, we ran three independent ensembles. We visually checked for convergence the marginal pdfs and distribution of log likelihoods of the three sampled chains. For all parameters, we computed Gelman and Rubin's potential scale reduction statistic R and effective sample size n_{eff} [35] using Simo Särkkä and Aki Vehtari's `psrf` function for MATLAB.[6] For each dataset and model, we looked at the largest R ($R_{\max}$) and smallest n_{eff} ($n_{\text{eff}\min}$) across parameters. Large values of R indicate convergence problems whereas values close to 1 suggest convergence. n_{eff} is an estimate of the actual number of independent samples in the chains; a few hundred independent samples are sufficient for a coarse approximation of the posterior [35]. Longer chains were run when suspicion of a convergence problem arose from any of these methods. Samples from independent ensembles were then combined (thinned, if necessary), yielding $1.5 \cdot 10^4$ posterior samples per dataset and model. In the end, average $R_{\max}$ (across datasets and models) was ~ 1.002 (range: $[1.000 - 1.035]$), suggesting good convergence. Average $n_{\text{eff}\min}$ was ~ 8881 (range: $[483 - 15059]$), suggesting that we had obtained a reasonable approximation of the posteriors.

4.4 Pareto smoothed importance sampling diagnostics

As our main metric of model comparison we computed the Bayesian leave-one-out cross-validation score (LOO) via Pareto-smoothed importance sampling (PSIS; [36, 37]); see Methods in the main text.

For a given trial $1 \leq i \leq N_{\text{trials}}$, with N_{trials} the total number of trials, the PSIS approximation may fail if the leave-one-out posterior differs too much from the full posterior. As a natural diagnostic, PSIS also returns for each trial the exponent k_i of the fitted Pareto distribution. If $k_i > 0.5$ the variance of the raw importance ratios distribution does not exist, and for $k_i > 1$ also the mean does not exist. In

[4]URL: `https://github.com/lacerbi/eissample`.
[5]URL: `https://github.com/lacerbi/vbgmm`.
[6]URL: `http://becs.aalto.fi/en/research/bayes/mcmcdiag/`.

the latter case, the variance of the PSIS estimate is still finite but may be large. In practice, Vehtari et al. suggest to double-check trials with $k_i > 0.7$ [37].

Across all our models and datasets, we found 2382 trials out of 1137100 with $k_i > 0.7$ (0.21%). We examined the problematic trials, finding that the issue was in almost all cases the discontinuity of the observer's decision rule. For all problematic trials the LOO_i scores were compatible with the values found for non-problematic trials, suggesting that the variance of the PSIS estimate was still within an acceptable range. We verified on a subset of subjects that the introduction a softmax with small spatial constant on the decision rule would remove the discontinuity and the problems with Pareto fitting, without significantly affecting the LOO_i itself.

4.5 Visualization of model fits

Let $\mathcal{O}(\mathcal{D})$ be a summary statistic of interest, that is an arbitrary function of a dataset $\mathcal{D}$ (e.g., the vestibular bias for a given bin of s_{vis} and visual reliability level, as per Fig 4 in the main paper). For a given model, we generated the posterior predictive distribution of the group mean of $\mathcal{O}$ by following this bootstrap procedure:

- For $m = 1, \ldots, M = 100$ iterations:
  - Generate a synthetic group of $n = 11$ subjects by taking n samples from the individual posterior distributions of the model parameters.
  - For each synthetic subject, generate a dataset $\mathcal{D}_i$ of simulated responses to the same trials experienced by the subject.
  - Compute the group mean of the summary statistic across synthetic subjects, $o_m = \frac{1}{n}\sum_{i=1}^{n} \mathcal{O}\left(\mathcal{D}_i\right)$.
- Compute mean and standard deviation of o_m, which correspond to group mean and SEM of the summary statistic.

The shaded areas shown in the model fits figures in the main text are the posterior predictive distributions (mean $\pm$ SEM) of the summary statistics of interest.

4.6 Model validation and recovery

We performed sanity checks and unit tests to verify the integrity of our code.

To test the implementation of our models, for a given observer (given model and parameter vector $\boldsymbol{\theta}$) we tested the data simulation code (functions that simulate responses; used e.g. to generate figures) against the log likelihood code (functions that compute the log likelihood of the data). For a number of subjects and models we verified that, at the maximum-likelihood solution, the log likelihood of the data approximated via simulation (by computing the probability of the responses via simple Monte Carlo) was $\sim$ equal to the log likelihood of the data computed numerically. This ensured that our simulation code matched the log likelihood code, being a sanity check for both.

We performed a model recovery analysis to validate the correctness of our analysis pipeline, and assess our ability to distinguish models of interest using all tasks ('joint fits'); see e.g. [13, 38]. For computational tractability, we restricted our analysis to six observer models: the most likely four models for each different causal inference strategy (to verify our ability to distinguish between strategies), and, for the most likely model, its variants along the prior and noise factors (to verify whether we can distinguish models along those axes). Thus, we consider the following models: Fix-X-E, Bay-X-E, Bay/FFu-X-I, Fix/FFu-C-I, Fix-X-I, Fix-C-E (see main text for a description). We generated synthetic datasets from each of these six models, for all three tasks jointly, using the same sets of stimuli that were originally displayed to the 11 subjects. For each subject, we took four randomly chosen posterior parameter vectors obtained via MCMC sampling (as described in Section 4.3), so as to ensure that the statistics of the

simulated responses were similar to those of the subjects. Following this procedure, we generated 264 datasets in total (6 generating models × 11 subjects × 4 posterior samples). We then fit all 6 models to each synthetic dataset, yielding 1584 fitting problems. For computational tractability, we only performed maximum likelihood estimation (see Section 4.2, with 50 restarts), as opposed to MCMC sampling, whose cost would be prohibitive for this number of fits. The analysis was otherwise exactly the same as that used for fitting the subject data. We then computed the fraction of times that a model was the 'best fitting' model for a given generating model, according to AICc (considering that AICc approximates LOO in the limit of large data).

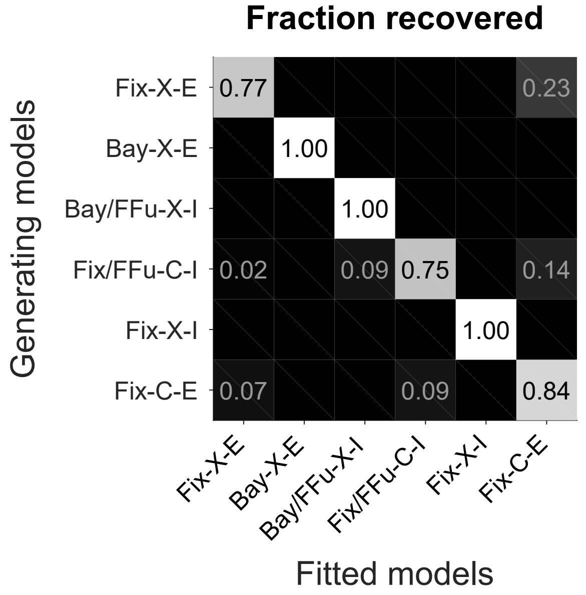

Model recovery analysis. Each square represents the fraction of datasets that were 'best' fitted from a model (columns), for a given generating model (rows), according to the AICc score. Bright shades of gray correspond to larger fractions. The bright diagonal indicates that the true generating model was, on average, the best-fitting model in all cases, leading to a successful model recovery.

We found that the true generating model was recovered correctly in 89.4% of the datasets on average (see above). This finding means that our models are distinguishable in a realistic setting, and at the same time validates the model fitting pipeline (as it would be unlikely to obtain a successful recovery in the presence of a substantial coding error). Since our model recovery method differs from the procedure used on subject data in the comparison metric (AICc via maximum-likelihood estimation, rather than LOO via MCMC), we verified on subject data that AICc and LOO scores were highly correlated across subjects [39]. The Spearman's rank correlation coefficient between the two metrics was larger than 0.99 for each of the sixteen models in the joint fits, providing strong evidence that results of our model recovery analysis would also transfer to the framework used for the subject data.

5 Absolute goodness of fit

In this section we describe a general method to compute absolute goodness of fit, largely based on the approach of [40].[7]

5.1 Computing the absolute goodness of fit

Let $\boldsymbol{X}$ be a dataset of discrete categorical data grouped in M independent batches with K classes each, such that X_{jk} is the number of observations for the j-th batch and the k-th class. We define $N_j = \sum_k X_{jk}$ the number of observations for the j-th batch.

We assume that observations are ordered and independent, such that the distribution of observations in each batch j is the product of N_j categorical distributions with parameters $\boldsymbol{p}_j = (p_{j1}, \ldots, p_{jK})$ (frequencies), such that the probability of the data is

$$p(\boldsymbol{X}) = \prod_{j=1}^{M} \prod_{k=1}^{K} p_{jk}^{X_{jk}}$$

with unknown vectors of frequencies $\boldsymbol{p}_j$.

We assume that we have a model of interest q that predicts frequencies q_{jk} for the observations, with $\sum_k q_{jk} = 1$ for $1 \leq j \leq M$. As a reference, we consider the chance model q^0 with frequencies $q^0_{jk} = 1/K$.

We define the *absolute goodness of fit* of q as

$$g\left(q\right) = 1 - \frac{\mathrm{KL}\left(p||q\right)}{\mathrm{KL}\left(p||q^0\right)}. \tag{S15}$$

where $\mathrm{KL}\left(p||q\right)$ is the Kullback-Leibler divergence (also known as relative entropy) between a 'true' distribution p and an 'approximating' distribution q.

Importantly, $g\left(q\right) = 0$ when a model performs at chance, and $g\left(q\right) \leq 1$, with $g\left(q\right) = 1$ only when the model matches the true distribution of the data. In other words, $g\left(q\right)$ represents the fractional information gain over chance. Note that $g\left(q\right)$ can be negative, in the unfortunate case that a model performs worse than chance.

As another important reference, we recommend to also compute the absolute goodness of fit $g\left(\bar{q}\right)$ of the *histogram model* $\bar{q}$, with frequencies defined from the empirical frequencies across batches as $\bar{q}_{jk} = \sum_{l=1}^{M} X_{lk}/N$, for $1 \leq j \leq M$ and $N = \sum_j N_j$. A comparison between $g\left(q\right)$ and $g\left(\bar{q}\right)$ is informative of how better the current model is than a simple histogram of categorical observations collapsed across batches. In some circumstances, the chance model can be a straw model, whereas the histogram model may represent a more sensible reference point.

In order to estimate Eq. S15, we need to compute the relative entropy $\mathrm{KL}\left(p||q\right)$ between the data and a given distribution q,

$$\begin{aligned} \mathrm{KL}\left(p||q\right) =& \mathbb{E}_p\left[\log p\right] - \mathbb{E}_p\left[\log q\right] \\ =& -H(p) + H(p,q) \end{aligned} \tag{S16}$$

where the first term is the (negative) entropy of the data, and the second term is called the cross-entropy between p and q. We will show in the following sections that the negative cross-entropy is approximated by the cross-validated log likelihood of the data, $\mathrm{LL}_{\mathrm{CV}}(q)$.

Combining Eq. S15 with our estimates of Eq. S16, we obtain

$$g\left(q\right) \equiv 1 - \frac{H(p) + \mathrm{LL}_{\mathrm{CV}}(q)}{H(p) + \mathrm{LL}_{\mathrm{chance}}(q)}. \tag{S17}$$

[7]URL: `https://github.com/lacerbi/gofit`.

We show next how to estimate the entropy of the data, and prove that the negative cross-entropy between p and q is approximated by the cross-validated log likelihood.

5.2 Entropy of the data

As noted in [40], the naïve plug-in estimator of the entropy of the data leads to a biased estimate of the entropy, and this bias can be substantial when the data are sparse (a few observations per batch). Instead, we use the Grassberger estimator of the entropy [41],

$$H(p) = \sum_{j=1}^{M} H(\boldsymbol{p}_j) \approx \sum_{j=1}^{M} N_j \hat{H}_G(\boldsymbol{X}_j) \tag{S18}$$

where the Grassberger estimator of the entropy per trial is defined as

$$\hat{H}_G(\boldsymbol{X}_j) = G\left(N_j\right) - \frac{1}{N_j} \sum_{k=1}^{K} X_{jk} G\left(X_{jk}\right) \tag{S19}$$

and $G\left(h\right)$ for $h \in \mathbb{N}$ are Grassberger's numbers defined as

$$G(0) = 0, \qquad G(h) = \psi\left(h\right) + \frac{1}{2}\left(-1\right)^h \left[\psi\left(\frac{h+1}{2}\right) - \psi\left(\frac{h}{2}\right)\right] \qquad \text{for } h > 0, \tag{S20}$$

where ψ is the digamma function.

That is, our estimate of the negative entropy is

$$-H(p) \approx -\sum_{j=1}^{M} N_j \left[G\left(N_j\right) - \frac{1}{N_j} \sum_{k=1}^{K} X_{jk} G(X_{jk})\right], \tag{S21}$$

which is the same as Eq. 21 in [40], when restricted to the binomial case ($K = 2$), and after correcting for a typo (N in the denominator of their equation should read as N_j).

5.3 Cross-entropy

The estimated cross-entropy is

$$\widehat{H}(p, q) = -\mathbb{E}_p\left[\log q\right] = -\sum_{j=1}^{M} N_j \mathbb{E}_{\boldsymbol{p}_j}\left[\log \boldsymbol{q}_j\right] = -\sum_{j=1}^{M} N_j \mathbb{E}_{\widehat{\boldsymbol{p}}_j}\left[\log \boldsymbol{q}_j\right] \tag{S22}$$

where in a slight abuse of notation we denoted with $\boldsymbol{p}_j$ (resp., $\boldsymbol{q}_j$) the categorical distributions associated to the data (resp., model) for the j-th batch. Crucially, since the expectations only involve q, $\widehat{p}_{jk} \equiv X_{jk}/N_j$ is an unbiased estimator of p_{jk}.

Eq. S22 becomes

$$
\begin{aligned}
-\sum_{j=1}^{M} N_j \mathbb{E}_{\widehat{\boldsymbol{p}}_j}\left[\log \boldsymbol{q}_j\right] &= -\sum_{j=1}^{M} N_j \mathbb{E}_{\widehat{\boldsymbol{p}}_j}\left[\log q_{j1}^{x_1}\cdots q_{jK}^{x_K}\right] \\
&= -\sum_{j=1}^{M} N_j \sum_{k=1}^{K} \mathbb{E}_{\widehat{\boldsymbol{p}}_j}\left[x_k\right]\log q_{jk} \\
&= -\sum_{j=1}^{M}\sum_{k=1}^{K} N_j \widehat{p}_{jk}\log q_{jk} \\
&= -\sum_{j=1}^{M}\sum_{k=1}^{K} X_{jk}\log q_{jk},
\end{aligned}
\tag{S23}
$$

which is the negative log likelihood of the model, $-\mathrm{LL}(q)$.

Note that typically we also need to estimate the model parameters, and computing Eq. S23 on the same dataset used to estimate parameters will yield a biased estimate of the log likelihood (see e.g., [42]). Shen and Ma suggest to obtain an independent estimate of the log likelihood of the model via cross-validation, $\mathrm{LL}_{\mathrm{CV}}$ [40]. According to their method, model parameters are estimated on half of the data, and the log likelihood of the model (and also the entropy of the data) is evaluated with the other half of the data. As an improvement over their method, we advocate to estimate the expected log likelihood via leave-one-out (LOO) cross-validation score obtained via MCMC [37]. This will produce an unbiased estimator of the expected log likelihood, and allows to use all the available data to obtain a more robust estimate of the relative entropy.

In conclusion, our estimate for the cross-entropy is

$$
\widehat{H}(p,q) = -\mathrm{LL}_{\mathrm{CV}}(q), \tag{S24}
$$

with $\mathrm{LL}_{\mathrm{CV}}(q)$ computed as the LOO score of the model, and it corresponds to Eq. 19 in [40].

6 LOO scores for all models

In this section we report tables of LOO scores for all models and subjects, which were used to perform group Bayesian Model Selection, the model comparison technique adopted in the main text. For each subject, LOO scores are shown relative to the LOO of the model with highest mean LOO across subject, which is printed in boldface. Models are ranked according to average LOO.

Summing (equivalently, averaging) LOO scores across subjects is a simple 'fixed-effect' model comparison analysis, in which all subjects are believed to belong to the same model. Results of the fixed-effect analysis differ in details from the group Bayesian Model Selection, but the overall qualitative findings are analogous.

6.1 Unity judgment task

**Model**	**S1**	**S2**	**S3**	**S4**	**S5**	**S6**	**S7**	**S8**	**S9**	**S10**	**S11**	**Mean ± SE**
**Bay-X-I**	**0.0**	**0.0**	**0.0**	**0.0**	**0.0**	**0.0**	**0.0**	**0.0**	**0.0**	**0.0**	**0.0**	**0.0 ± 0.0**
Bay-X-E	−22.1	4.5	−12.7	−29.7	22.2	−24.7	−1.8	−2.6	1.7	35.9	−0.4	−2.7 ± 5.9
Fix	−31.6	12.5	−12.9	0.7	−12.4	−18.8	1.8	12.3	−2.8	10.2	−4.8	−4.2 ± 4.2
Bay-C-I	−0.3	4.6	0.4	−11.7	−11.9	2.2	−0.4	−0.8	−2.8	−25.6	−1.8	−4.4 ± 2.6
Fix-C	−30.6	13.2	−10.5	2.3	−21.1	−18.0	1.1	14.4	−2.6	−29.0	−7.6	−8.0 ± 4.7
Bay-C-E	−26.4	−18.7	−14.2	−29.8	16.0	−41.9	−1.6	−17.0	−1.9	12.5	−2.9	−11.4 ± 5.4
SFu	−272.4	−119.9	−245.8	−122.5	−112.1	−154.5	−272.4	−120.9	−250.2	−122.0	−117.5	−173.7 ± 21.1

6.2 Bimodal inertial discrimination task

Model	S1	S2	S3	S4	S5	S6	S7	S8	S9	S10	S11	Mean ± SE
**Bay-X-E**	**0.0**	**0.0**	**0.0**	**0.0**	**0.0**	**0.0**	**0.0**	**0.0**	**0.0**	**0.0**	**0.0**	**0.0 ± 0.0**
Fix-X-E	−0.9	0.5	−2.2	−13.0	−0.8	0.8	−0.6	0.4	0.3	3.4	1.5	−1.0 ± 1.3
FFu-X-I	−0.7	0.9	−3.5	−11.3	0.4	1.3	1.2	1.6	1.5	−12.3	1.1	−1.8 ± 1.6
Fix-X-I	−0.9	2.0	−3.2	−11.5	0.6	1.3	0.6	0.0	0.7	−12.5	1.1	−2.0 ± 1.6
FFu-X-E	−0.2	0.9	−3.6	−10.2	0.6	1.5	0.9	1.5	1.4	−18.8	1.2	−2.3 ± 2.0
Fix-C-E	−9.8	−3.7	0.1	−18.7	−0.9	−2.5	−7.1	1.1	−2.3	3.9	0.4	−3.6 ± 1.9
Bay-C-E	−10.5	0.3	−0.6	−5.7	0.6	−1.9	−11.8	0.1	−1.8	−5.4	−3.2	−3.6 ± 1.3
Bay-X-I	−3.1	−2.6	−5.7	−13.0	−1.6	−1.5	0.2	−0.8	0.2	−15.5	1.4	−3.8 ± 1.7
FFu-C-E	−20.1	−22.1	−9.9	−34.7	−14.8	−21.9	−31.9	−6.0	−2.4	−57.7	−2.1	−20.3 ± 5.0
FFu-C-I	−20.2	−22.1	−9.9	−34.8	−14.8	−21.8	−31.9	−6.0	−2.6	−57.7	−2.2	−20.3 ± 5.0
Fix-C-I	−20.2	−22.1	−9.9	−34.8	−14.8	−21.9	−30.6	−6.8	−3.1	−57.8	−2.3	−20.4 ± 4.9
Bay-C-I	−19.6	−21.6	−10.4	−34.7	−15.9	−22.7	−32.3	−6.2	−2.8	−58.2	−2.8	−20.6 ± 5.0

6.3 Joint fits

Model	S1	S2	S3	S4	S5	S6	S7	S8	S9	S10	S11	Mean ± SE
**Fix-X-E**	**0.0**	**0.0**	**0.0**	**0.0**	**0.0**	**0.0**	**0.0**	**0.0**	**0.0**	**0.0**	**0.0**	**0.0 ± 0.0**
Fix-X-I	7.3	−7.2	−4.4	31.9	−16.1	23.7	−40.6	−26.6	−38.6	−20.4	2.4	−8.0 ± 7.1
Fix/FFu-X-E	−14.8	−19.7	−15.4	4.8	−3.4	0.5	−42.6	−4.3	−14.8	−8.9	2.4	−10.6 ± 4.0
Fix-C-E	0.4	−9.2	−1.9	−14.3	−41.9	−7.9	−3.5	0.9	−6.5	−46.7	−10.7	−12.8 ± 4.9
Fix/FFu-X-I	−26.6	−19.7	−22.5	4.8	−2.4	0.5	−52.4	−4.2	−59.7	−2.9	2.3	−16.6 ± 6.7
Bay-X-E	17.1	−34.2	−6.2	−31.3	−25.8	−20.6	−9.5	−128.6	12.7	12.3	−6.2	−20.0 ± 12.1
Bay/FFu-X-E	−20.8	−39.0	−25.3	0.5	10.8	−14.9	−42.9	−127.0	−40.8	3.7	1.2	−26.8 ± 11.6
Fix/FFu-C-E	−14.2	−21.0	−17.9	−20.5	−47.6	−6.0	−44.5	−3.3	−19.0	−103.6	−8.5	−27.8 ± 8.7
Fix-C-I	−3.6	−32.0	−15.8	−14.1	−59.5	2.1	−85.5	−25.2	−59.0	−94.9	−9.4	−36.1 ± 10.1
Fix/FFu-C-I	−25.6	−21.1	−22.0	−20.6	−47.7	−6.1	−86.0	−3.4	−59.1	−103.7	−8.6	−36.7 ± 10.1
Bay-C-E	2.7	−73.1	−29.5	−44.2	−33.6	−74.9	−16.1	−191.4	−6.7	−12.3	−26.4	−45.9 ± 16.4
Bay/FFu-C-E	−36.4	−77.3	−47.1	−31.0	−15.4	−45.3	−90.7	−206.7	−72.9	−74.3	−11.8	−64.4 ± 16.2
Bay-X-I	−356.3	−128.2	−193.6	−204.0	−91.3	−35.6	−177.3	−235.6	−298.7	−105.2	−6.3	−166.6 ± 32.2
Bay-C-I	−462.0	−222.2	−318.1	−231.3	−158.8	−77.6	−319.6	−338.0	−488.1	−259.2	−51.2	−266.0 ± 42.1
Bay/FFu-X-I	−872.8	−416.4	−544.9	−589.5	−304.5	−424.8	−555.4	−397.0	−593.0	−272.0	−53.7	−456.7 ± 64.2
Bay/FFu-C-I	−888.7	−445.3	−556.3	−611.2	−340.1	−441.8	−551.3	−396.2	−625.7	−351.2	−69.8	−479.8 ± 62.6

Supplemental References

1. Wozny DR, Beierholm UR, Shams L. Human trimodal perception follows optimal statistical inference. Journal of vision. 2008;8(3):1–24.

2. Wallace MT, Roberson G, Hairston WD, Stein BE, Vaughan JW, Schirillo JA. Unifying multisensory signals across time and space. Experimental Brain Research. 2004;158(2):252–258.

3. Rohe T, Noppeney U. Sensory reliability shapes perceptual inference via two mechanisms. Journal of Vision. 2015;15(5):1–22.

4. Gu Y, Fetsch CR, Adeyemo B, DeAngelis GC, Angelaki DE. Decoding of MSTd population activity accounts for variations in the precision of heading perception. Neuron. 2010;66(4):596–609.

5. Cuturi LF, MacNeilage PR. Systematic biases in human heading estimation. PLoS ONE. 2013;8(2):e56862.

6. Stocker AA, Simoncelli EP. Noise characteristics and prior expectations in human visual speed perception. Nature Neuroscience. 2006;9(4):578–585.

7. Girshick AR, Landy MS, Simoncelli EP. Cardinal rules: Visual orientation perception reflects knowledge of environmental statistics. Nature Neuroscience. 2011;14(7):926–932.

8. Ganguli D, Simoncelli EP. Efficient sensory encoding and Bayesian inference with heterogeneous neural populations. Neural computation. 2014;26(10):2103–2134.

9. Wei XX, Stocker AA. A Bayesian observer model constrained by efficient coding can explain 'anti-Bayesian' percepts. Nature neuroscience. 2015;18(10):1509.

10. Ernst MO, Banks MS. Humans integrate visual and haptic information in a statistically optimal fashion. Nature. 2002;415(6870):429–433.

11. Alais D, Burr D. The ventriloquist effect results from near-optimal bimodal integration. Current Biology. 2004;14(3):257–262.

12. Fetsch CR, Turner AH, DeAngelis GC, Angelaki DE. Dynamic reweighting of visual and vestibular cues during self-motion perception. The Journal of Neuroscience. 2009;29(49):15601–15612.

13. Acerbi L, Ma WJ, Vijayakumar S. A Framework for Testing Identifiability of Bayesian Models of Perception. In: Advances in Neural Information Processing Systems 27. Curran Associates, Inc.; 2014. p. 1026–1034.

14. Petzschner FH, Glasauer S. Iterative Bayesian estimation as an explanation for range and regression effects: A study on human path integration. The Journal of Neuroscience. 2011;31(47):17220–17229.

15. Odegaard B, Shams L. The Brain's Tendency to Bind Audiovisual Signals Is Stable but Not General. Psychological Science. 2016;27(4):583–591. doi:10.1177/0956797616628860.

16. Qamar AT, Cotton RJ, George RG, Beck JM, Prezhdo E, Laudano A, et al. Trial-to-trial, uncertainty-based adjustment of decision boundaries in visual categorization. Proceedings of the National Academy of Sciences. 2013;110(50):20332–20337.

17. Ma WJ. Organizing probabilistic models of perception. Trends in Cognitive Sciences. 2012;16(10):511–518.

18. Rohe T, Noppeney U. Cortical hierarchies perform Bayesian causal inference in multisensory perception. PLoS Biol. 2015;13(2):e1002073.

19. Wichmann FA, Hill NJ. The psychometric function: I. Fitting, sampling, and goodness of fit. Percept Psychophys. 2001;63(8):1293–1313.

20. de Winkel KN, Katliar M, Diers D, Buelthoff HH. What's Up: an assessment of Causal Inference in the Perception of Verticality. bioRxiv. 2017; p. 189985.

21. Stevens SS. On the psychophysical law. Psychological review. 1957;64(3):153.

22. Crane BT. Direction specific biases in human visual and vestibular heading perception. PLoS ONE. 2012;7(12):e51383.

23. de Winkel KN, Katliar M, Bülthoff HH. Forced fusion in multisensory heading estimation. PLoS ONE. 2015;10(5):e0127104.

24. de Winkel KN, Katliar M, Bülthoff HH. Causal Inference in Multisensory Heading Estimation. PLoS ONE. 2017;12(1):e0169676.

25. Körding KP, Beierholm U, Ma WJ, Quartz S, Tenenbaum JB, Shams L. Causal inference in multisensory perception. PLoS ONE. 2007;2(9):e943.

26. Press WH, Flannery BP, Teukolsky SA, Vetterling WT. Numerical recipes 3rd edition: The art of scientific computing. Cambridge University Press; 2007.

27. Acerbi L, Ma WJ. Practical Bayesian Optimization for Model Fitting with Bayesian Adaptive Direct Search. In: Advances in Neural Information Processing Systems 30; 2017. p. 1836–1846.

28. Audet C, Dennis Jr JE. Mesh adaptive direct search algorithms for constrained optimization. SIAM Journal on Optimization. 2006;17(1):188–217.

29. Neal RM. Slice sampling. Annals of Statistics. 2003;31(3):705–741.

30. Gilks WR, Roberts GO, George EI. Adaptive direction sampling. The Statistician. 1994;43(1):179–189.

31. Foreman-Mackey D, Hogg DW, Lang D, Goodman J. emcee: The MCMC hammer. Publications of the Astronomical Society of the Pacific. 2013;125(925):306.

32. Bishop CM. Pattern recognition and machine learning. Springer; 2006.

33. Andrieu C, Thoms J. A tutorial on adaptive MCMC. Statistics and Computing. 2008;18(4):343–373.

34. MacKay DJ. Information theory, inference and learning algorithms. Cambridge university press; 2003.

35. Gelman A, Carlin JB, Stern HS, Dunson DB, Vehtari A, Rubin DB. Bayesian data analysis (3rd edition). CRC Press; 2013.

36. Vehtari A, Gelman A, Gabry J. Pareto smoothed importance sampling. arXiv preprint arXiv:150702646. 2015;.

37. Vehtari A, Gelman A, Gabry J. Practical Bayesian model evaluation using leave-one-out cross-validation and WAIC. Statistics and Computing. 2016; p. 1–20.

38. van den Berg R, Awh E, Ma WJ. Factorial comparison of working memory models. Psychological Review. 2014;121(1):124–149.

39. Adler WT, Ma WJ. Comparing Bayesian and non-Bayesian accounts of human confidence reports. bioRxiv. 2016;doi:10.1101/093203.

40. Shen S, Ma WJ. A detailed comparison of optimality and simplicity in perceptual decision making. Psychological Review. 2016;123(4):452–480.

41. Grassberger P. Entropy estimates from insufficient samplings. arXiv preprint physics/0307138. 2003;.

42. Burnham KP, Anderson DR. Model selection and multimodel inference: A practical information-theoretic approach. Springer Science & Business Media; 2003.
